# Characterization of the complete mitochondrial genome of *Desmaulus extinctorium* (Littorinimorpha, Calyptraeoidea, Calyptraeidae) and molecular phylogeny of Littorinimorpha

**DOI:** 10.1371/journal.pone.0301389

**Published:** 2024-03-28

**Authors:** Yanwen Ma, Biqi Zheng, Jiji Li, Wei Meng, Kaida Xu, Yingying Ye

**Affiliations:** 1 National Engineering Research Center for Marine Aquaculture, Zhejiang Ocean University, Zhoushan, 316022, China; 2 Department of Natural Resources, Ningde Marine Center, Ningde, 352000, China; 3 Key Laboratory of Sustainable Utilization of Technology Research for Fisheries Resources of Zhejiang Province, Zhejiang Marine Fisheries Research Institute, Scientific Observing and Experimental Station of Fishery Resources for Key Fishing Grounds, Ministry of Agriculture and Rural Affairs of China, Zhoushan, 316021, China; University of Saskatchewan College of Agriculture and Bioresources, CANADA

## Abstract

For the purpose of determining the placement of Calyptraeidae within the Littorinimorpha, we hereby furnish a thorough analysis of the mitochondrial genome (mitogenome) sequence of *Desmaulus extinctorium*. This mitogenome spans 16,605 base pairs and encompasses the entire set of 37 genes, including 13 PCGs, 22 tRNAs and two rRNAs, with an evident AT bias. Notably, *tRNA*^*Ser1*^ and *tRNA*^*Ser2*^ lack dihydrouracil (DHU) arms, resulting in an inability to form a secondary structure. Similarly, *tRNA*^*Ala*^ lacks a TΨC arm, rendering it incapable of forming a secondary structure. In contrast, the remaining tRNAs demonstrate a characteristic secondary structure reminiscent of a cloverleaf. A comparison with ancestral gastropods reveals distinct differences in three gene clusters (or genes), encompassing 15 tRNAs and eight PCGs. Notably, inversions and translocations represent the major types of rearrangements observed in *D*. *extinctorium*. Phylogenetic analysis demonstrates robust support for a monophyletic grouping of all Littorinimorpha species, with *D*. *extinctorium* representing a distinct Calyptraeoidea clade. In summary, this investigation provides the first complete mitochondrial dataset for a species of the Calyptraeidae, thus providing novel insights into the phylogenetic relationships within the Littorinimorpha.

## Introduction

Mitochondria are double—membrane—coated organelles found in most eukaryotes. Although most of a cell’s DNA is contained in the nucleus, mitochondria have their own genome, known as the mitogenome. Attributable to its profoundly conserved characteristics, absence of extensive recombination, maternal inheritance, and elevated mutation rate [1–3], the mitogenome has found extensive utility in the realms of comparative and evolutionary genomics [4], species identification, population genetics [5], molecular evolution, and phylogenetic relationships [6,7]. In particular, phylogeny based on complete mitochondrial genomes have demonstrated improved resolution compared to phylogenetic trees inferred from partial gene fragments such as *COI* and *16S rRNA* [8]. In recent years, mitochondrial genome sequencing and amplification techniques have rapidly developed, and mitochondrial genomes have been extensively utilized to reconstruct phylogenetic trees of different gastropods. For instance, Yang et al [9] sequenced the complete mitochondrial genomes of nine Nassariidae species and compared them with eight previously reported Nassariidae genomes, identifying the phylogenetic placement of these nine species within the gastropod clade. Genetic distance analysis and phylogenetic analysis both supported the distant relationship of *Nassarius jacksonianus* and *Nassarius acuticostus* to other *Nassarius* species. Furthermore, Yang et al [10] sequenced the complete mitochondrial genomes of two nassarids (Neogastropoda: Nassariidae: *Nassarius*), *Nassarius glans* and *Nassarius siquijorensis*, identifying the phylogenetic positions of these two species within *Nassarius*. In addition, Lee et al [11] reported the complete mitochondrial genome of *Semisulcospira gottschei* (Gastropoda: Caenogastropoda) and identified its phylogenetic relationship within Caenogastropoda. The study revealed that *Semisulcospira gottschei* is the closest relative to *Semisulcospira coreana*, and it was classified within the family Cerithioidea.

*Desmaulus extinctorium* is a marine snail that inhabits sandy substrates ranging from low intertidal to several metres subtidally. It belongs to the class Gastropoda, subclass Caenogastropoda, order Littorinimorpha, superfamily Calyptraeoidea, family Calyptraeidae, genus *Desmaulu*s. *Desmaulus extinctorium* is abundant in southern China and Hongkong, with a widespread presence in the Indo-West Pacific region as well [12]. Previous research on this family has predominantly focused on morphology and growth [13–15]. Calyptraeid gastropods are known for their taxonomic challenges stemming from their simple, phenotypically variable shells [16]. As such, only a few studies have explored the phylogenetic analysis of this family. For instance, Cunha et al [17] conducted sequencing on a segment of the mitochondrial genome from the calyptraeoidean species *Calyptraea chinensis*, which belongs to the Littorinimorpha. Phylogenetic investigations have revealed that the Littorinimorpha does not form a monophyletic cluster. Meanwhile, Collin [18] examined how development modes influence the phylogeography and population dynamics of North Atlantic *Crepidula* (Gastropoda: Calyptraeidae). She created haplotype trees for each clade using 640 bp *COI* sequences. Examination of both the tree topology and AMOVA revealed that species undergoing direct development (hatching as benthic juveniles) displayed a more conspicuous population structure in comparison to those species undergoing planktonic development. Prior to our study, a complete mitochondrial genome of Calyptraeidae had not been uploaded to GenBank.

Littorinimorpha is a substantial order within Caenogastropoda (Class Gastropoda), encompassing 16 superfamilies according to the WoRMS database. Among marine snails, Caenogastropoda stands as the dominant group in terms of species numbers, diversity of habitats, ecological importance and behaviors. The current classification within Littorinimorpha was mainly established by Bouchet and Rocroi [19]. While Colgan et al [20] conducted an exhaustive phylogenetic investigation of Caenogastropoda, the interrelationships among families and superfamilies within the Caenogastropoda clade remain predominantly unresolved. The monophyly of both Littorinimorpha and Neogastropoda has been a topic of ongoing debate [21]. Cunha et al [22] conducted the sequencing of complete mitochondrial genomes for seven previously unanalyzed gastropod species. Subsequent phylogenetic analysis led to the rejection of the monophyletic status of Neogastropoda, attributed to the incorporation of Littorinimorpha lineages within this cluster. Additionally, Zhao et al. [23] sequenced the complete mitochondrial genomes of intermediate host snails for *Schistosoma* and performed a phylogenetic analysis, revealing that neither Neogastopoda nor Littorinimorpha were monophyletic groups. Consequently, further research is necessary to refine the phylogenetic relationship within Caenogastropoda. Riedel [24] established the superfamily Ficoidea, separate from the Tonnoidea, but based on the sequencing of the complete mitochondrial genome of *Ficus variegata* Wang et al. [25] demonstrated that it fits within the Tonnoidea. And then, Jiang et al [26] reconstructed the phylogenetic tree of Littorinimorpha by sequencing the complete mitochondrial genome of two species in the Stromboidea. The findings provided evidence for the existence of three significant clades within Littorinimorpha: 1) Stromboidea, Tonnoidea, Littorinoidea, and Naticoidea, 2) Rissooidea alongside Truncatelloidea, and 3) Vermetoidea.

In this investigation, we have accomplished the comprehensive sequencing of the mitogenome for *D*. *extinctorium*. Furthermore, an elucidation of the gene structure within the mitogenome of *D*. *extinctorium* has been presented, coupled with a phylogenetic scrutiny encompassing 51 species from the Littorinimorpha taxon. This analysis is predicated upon the nucleotide sequences of 13 PCGs. As an outcome of this study, there has been an augmentation of the mitochondrial genome repertoire for Littorinimorpha, along with the provision of data requisite for subsequent phylogenetic assessments within the Littorinimorpha clade.

## Materials and methods

### Sampling and DNA extraction

We obtained a specimen of *D*. *extinctorium* from Ningde, Fujian Province, China (27°04′812N, 120°24′158″E). The initial morphological classification of these samples involved expert consultation with taxonomists at Zhejiang Ocean University’s Marine Biological Museum. After collection, the specimen was rapidly submerged in absolute ethanol and stored at -20°C. To confirm its classification, we relied on morphological traits, and we preserved fresh tissues in absolute ethanol before DNA extraction. We used the salt-extraction technique [27] to isolate complete genomic DNA, which was then stored at -20°C.

### Genome sequencing, assembly and annotation

The mitogenomes of *D*. *extinctorium* were sequenced by Origin gene Co. Ltd., situated in Shanghai, China, employing the Illumina HiSeq X Ten sequencing platform. HiSeq X Ten libraries were prepared, incorporating an insert size ranging from 300 to 500 base pairs, sourced from genomic DNA samples. Each library yielded approximately 10 gigabases of raw data. Preprocessing procedures encompassed the elimination of low-quality reads, adapters, sequences containing high proportions of ambiguous bases ("N" bases), and those with a length below 25 base pairs. For assembly, the NOVOPlasty software [28] (accessible at https://github.com/ndierckx/NOVOPlasty) was utilized. Annotation and manual refinement of the assembly were performed with reference to established mitogenome datasets. De novo assembled mitogenomes were generated using MITOS tools [29] (accessed through the MITOS Web Server at uni-leipzig.de). Validation of sequence accuracy was achieved through alignment against mitochondrial genes of other Calyptraeoidea species, complemented by confirmation via the COI barcode sequence and NCBI BLAST searches [30].

Reads were reconstructed using a de novo assembly program, and subsequent annotation of complete mitogenomes was conducted using Sequin version 16.0. The mitogenome map of *D*. *extinctorium* was visualized utilizing the online tool Poksee (accessible at https://proksee.ca) [31]. Secondary structures of tRNA genes were forecasted and illustrated through the MITOS Web Server. To gain insights into coding sequence characteristics, relative synonymous codon usage (RSCU) values and substitution saturation for the 13 protein-coding genes (PCGs) were computed utilizing DAMBE 5. Subsequent analysis of these values was executed using MEGA 7 [32]. Additionally, base compositional disparities and strand asymmetry among samples were assessed by evaluating GC-skews and AT-skews. These parameters were calculated using the following formulas: AT-skew = [A−T]/[A+T] and GC skew = [G−C]/[G+C]. Substitution saturation for the 13 PCGs was quantified using DAMBE 5 [33].

### Gene order analysis

In addition to the mitogenomes sequenced in this study, we obtained an additional 51 complete mitogenomes of Littorinimorpha from GenBank (Table 1) for comparative analyses. The gene arrangements of all 51 mitogenomes were compared with the ancestral Gastropoda, with the aim of identifying potential novel gene orders that have not been reported in previous studies. To ensure that observed gene order differences were not caused by mis-annotations, any mitogenomes in Littorinimorpha that deviated from the ancestral pattern underwent re-annotation using MITOS [29].

**Table 1:** List of species of Littorinimorpha analysed in this study and their GenBank accession numbers.

Superfamily	Family	Species	Accession no.	Size(bp)
Stromboidea	Strombidae	*Aliger gigas*[34]	MZ157283	15460
		*Conomurex luhuanus*[35]	NC_035726	15799
		*Harpago chiragra*[36]	MN885884	16404
		*Laevistrombus canarium*[37]	MT937083	15626
		*Lambis lambis*[36]	MH115428	15481
		*Strombus pugilis*[38]	MW244819	15809
		*Tridentarius dentatus*[38]	MW244820	15500
	Aporrhaidae	*Aporrhais serresiana*[38]	MW244817	15455
	Struthiolariidae	*Struthiolaria papulosa*[38]	MW244818	15475
	Seraphsidae	*Terebellum terebellum*[38]	MW244821	15478
	Rostellariidae	*Tibia fusus*	NC_065371	16083
		*Varicospira cancellata*[38]	MW244822	15864
	Xenophoridae	*Xenophora japonica*[38]	MW244823	15684
Truncatelloidea	Amnicolidae	*Baicalia turriformis*[39]	NC_035869	15127
		*Godlewskia godlewskii*[39]	NC_035870	15224
		*Maackia herderiana*[39]	NC_035871	15154
	Pomatiopsidae	*Oncomelania hupensis*	NC_013073	15182
		*Tricula hortensis*	NC_013833	15179
	Tateidae	*Potamopyrgus antipodarum*[40]	NC_070577	16846
		*Potamopyrgus estuarinus*[40]	NC_070576	16701
Tonnoidea	Bursidae	*Bufonaria rana*[41]	MT408027	15510
	Charoniidae	*Charonia lampas*	KU237290	15330
		*Charonia tritonis*	MT043269	15346
	Cassidae	*Galeodea echinophora*[21]	NC_028003	15388
	Cymatiidae	*Monoplex parthenopeus*[17]	NC_013247	15270
Naticoidea	Naticidae	*Cryptonatica andoi*[42]	NC_046598	15302
		*Cryptonatica janthostoma*[42]	NC_046704	15235
		*Euspira gilva*[42]	NC_046593	15315
		*Euspira pila*[42]	NC_046703	15244
		*Glossaulax reiniana*[43]	NC_041162	15254
		*Mammilla mammata*[42]	NC_046597	15319
		*Mammilla kurodai*[42]	NC_046596	15309
		*Naticarius hebraeus*[21]	NC_028002	15384
		*Neverita didyma*[42]	NC_046594	15629
		*Notocochlis gualteriana*[42]	NC_046705	15176
		*Paratectonatica tigrina*[42]	NC_050661	15201
		*Polinices sagamiensis*[42]	NC_046595	15383
		*Tanea lineata*[42]	NC_050662	15156
Cypraeoidea	Cypraeidae	*Cypraea tigris*[44]	MK783263	16177
		*Erronea errones*	NC_066082	15422
Vermetoidea	Vermetidae	*Dendropoma gregarium*[45]	NC_014580	15641
		*Eualetes tulipa*[45]	NC_014585	15078
		*Thylacodes squamigerus*[45]	NC_014588	15544
Ficoidea	Ficidae	*Ficus variegata*	NC_056153	15736
Littorinoidea	Littorinidae	*Littoraria ardouiniana*	NC_066085	16261
		*Littoraria intermedia*	NC_064397	16194
		*Littoraria melanostoma*	NC_064398	16149
		*Littoraria sinensis*[46]	MN496138	16420
		*Littorina brevicula*[47]	NC_050987	16356
		*Littorina saxatilis*	NC_030595	16887
		*Melarhaphe neritoides*[48]	MH119311	15676
Calyptraeoidea	Calyptraeidae	*Desmaulus extinctorium*	OQ511529	16572
Outgroup		*Donax variegatus*[49]	NC_035986	17195
		*Donax vittatus*[49]	NC_035987	17070

### Phylogenetic analysis

Exploring the evolutionary relationships within the Littorinimorpha clade involved an analysis of 13 PCGs. These genes were sourced from a comprehensive dataset that included 51 complete mitogenome sequences. The mitogenome sequences were retrieved from the GenBank database (https://www.ncbi.nlm.nih.gov/genbank/). To provide additional context, two species from the Donacidae family were also included as representatives of the outgroup. The assessment of phylogenetic relationships utilized both Maximum Likelihood (ML) and Bayesian Inference (BI) methodologies [50–52].

The ML analysis, carried out with IQ-TREE 1.6.2, involved 1000 ultrafast likelihood bootstrap replicates. The choice of optimal models was guided by the Bayesian Information Criterion (BIC), leading to the adoption of the GTR + F + R6 model for each partition. We conducted Bayesian Inference (BI) analyses using the MrBayes 3.2 software, and model selection was facilitated by MrMTgui [53], a tool that connects PAUP, ModelTest, and MrModelTest across different platforms. For model selection, we chose the best-fit model (GTR + I + G) based on AIC results obtained from MrModelTest 2.3 [54]. Bayesian analyses were then performed in MrBayes, utilizing parameter values from either MrModelTest or ModelTest (nst = 6, rates = invgamma) [55]. The Bayesian analyses utilized Markov Chain Monte Carlo (MCMC) sampling, involving two independent runs of three hot chains and one cold chain. These chains ran simultaneously for 2,000,000 generations, with sampling intervals set at 1000 steps and a relative burn-in rate of 25%. We assessed the convergence of independent runs by examining the mean standard deviation of split frequencies (< 0.01). Finally, the resulting phylogenetic trees were visualized and edited using Figure Tree v.1.4.3 software [56].

## Results discussion

### Genome structure and composition

**Fig 1:**
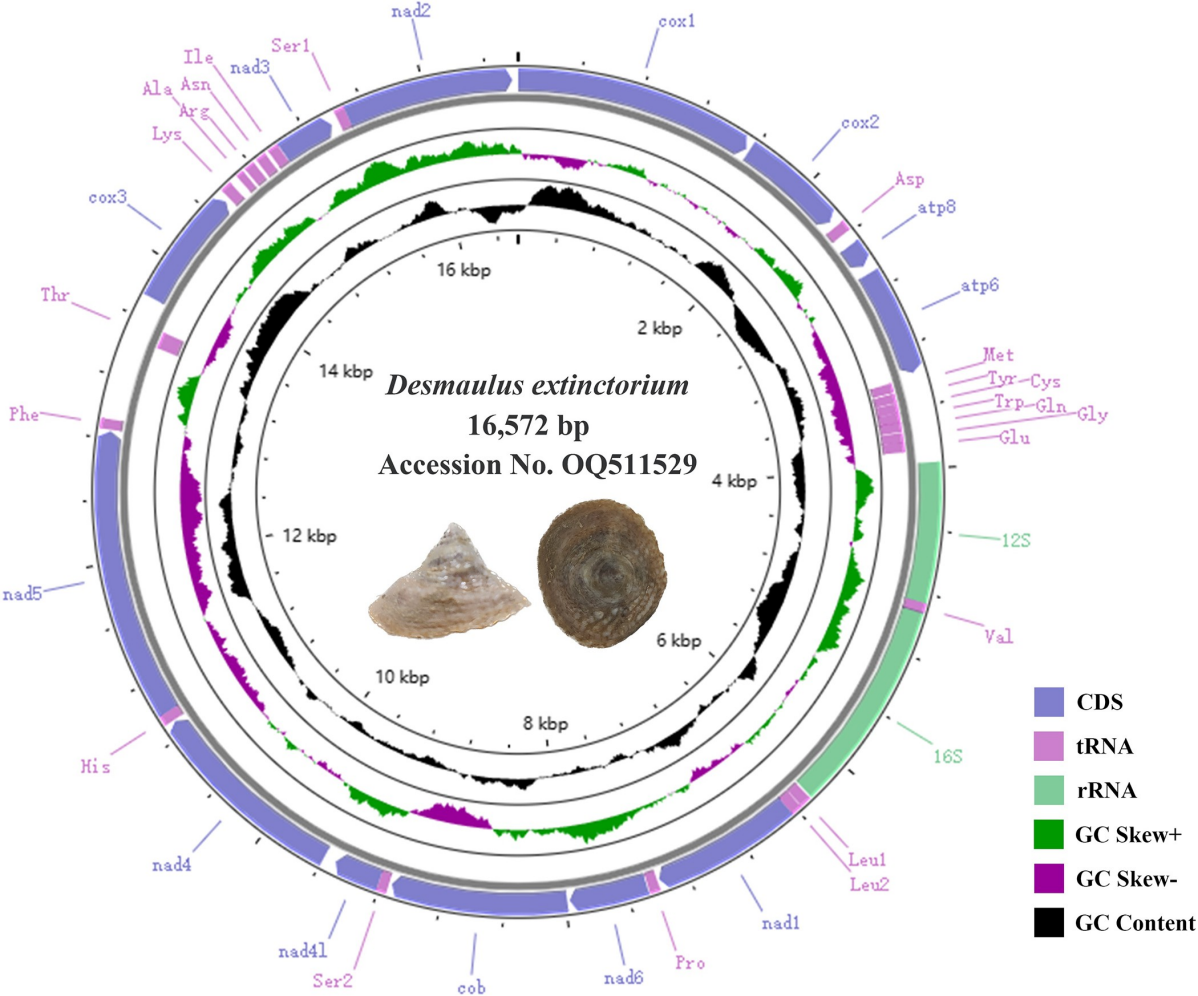
Maps of the mitochondrial genomes of *D*. *extinctorium*.

The complete mitogenome sequence of *D*. *extinctorium* constitutes a prototypical closed-circular molecule spanning 16,605 bp in length (GenBank accession number OQ511529). This genome encompasses a total of 37 genes, comprising 13 protein-coding genes (PCGs), 22 transfer RNAs (tRNAs), two ribosomal RNAs (*16S rRNA* and *12S rRNA*), and a concise non-coding region. This structural arrangement aligns consistently with the composition observed in the majority of previously investigated mollusks [57–59]. All these genes have been discerned and are depicted in Fig 1 and Table 2. Among the 37 genes, the majority are localized on the heavy (H-) strand, except for eight tRNAs (*tRNA-Phe*, *His*, *Pro*, *Leu*, *Val*, *Gln*, *Cys*, and *Tyr*). (Fig 1. Maps of the mitochondrial genomes of *D*. *extinctorium*.)

**Table 2:** Mitochondrial genome organization of *D*. *extinctorium*.

Gene	Direction	Position	Length/bp		Start/Stop codon	Intergenic Nucleotide(bp)	Anticodon
*COX1*	H	1	1551	1551	ATT/TAA	22	
*COX2*	H	1574	2266	693	ATG/TAA	46	
*tRNA* ^*Asp*^	H	2313	2382	70		82	GTC
*ATP8*	H	2465	2623	159	ATG/TAA	57	
*ATP6*	H	2681	3376	696	ATG/TAA	26	
*tRNA* ^*Met*^	L	3403	3468	66		8	CAT
*tRNA* ^*Tyr*^	L	3477	3542	66		4	GTA
*tRNA* ^*Cys*^	L	3547	3612	66		0	GCA
*tRNA* ^*Trp*^	L	3613	3679	67		-2	TCA
*tRNA* ^*Gln*^	L	3678	3743	66		4	TTG
*tRNA* ^*Gly*^	L	3748	3813	66		-1	TCC
*tRNA* ^*Glu*^	L	3813	3882	70		80	TTC
*12S rRNA*	H	3963	4858	896		-1	
*tRNA* ^*Val*^	H	4858	4925	68		-10	TAC
*16S rRNA*	H	4916	6279	1364		13	
*tRNA* ^*Leu1*^	H	6293	6364	72		4	TAG
*tRNA* ^*Leu2*^	H	6369	6438	70		0	TAA
*NAD1*	H	6439	7383	945	ATG/TAA	12	
*tRNA* ^*Pro*^	H	7396	7463	68		6	TGG
*NAD6*	H	7470	7973	504	ATG/TAA	16	
*Cytb*	H	7990	9129	1140	ATG/TAA	17	
*tRNA* ^*Ser2*^	H	9147	9212	66		0	TGA
*NAD4l*	H	9213	9515	303	ATG/TAG	80	
*NAD4*	H	9596	10900	1305	ATG/TAA	10	
*tRNA* ^*His*^	H	10911	10976	66		0	GTG
*NAD5*	H	10977	12848	1872	ATG/TAG	10	
*tRNA* ^*Phe*^	H	12859	12924	66		12	GAA
*tRNA* ^*Thr*^	L	13573	13640	68		104	TGT
*COX3*	H	13745	14524	780	ATG/TAA	29	
*tRNA* ^*Lys*^	H	14554	14628	75		13	TTT
*tRNA* ^*Ala*^	H	14684	14734	51		17	TGC
*tRNA* ^*Arg*^	H	14752	14821	70		21	TCG
*tRNA* ^*Asn*^	H	14843	14912	70		23	GTT
*tRNA* ^*Ile*^	H	14936	15005	70		0	GAT
*NAD3*	H	15010	15366	357	ATG/TAG	1	
*tRNA* ^*Ser1*^	H	15416	15483	68		2	GCT
*NAD2*	H	15484	16572	1089	ATG/TAA	36	

The longest gene, *ND5*, stretches across 1872 base pairs, whereas the shortest is *tRNA*^*Ala*^, comprising a mere 51 base pairs. The *D*. *extinctorium* mitogenome comprises four regions displaying overlap. Of these, one involves a 10 bp overlap with *tRNA*^*Val*^, and the remaining three exhibit overlaps shorter than 10 bp with *tRNA*^*Trp*^ (2 bp), *tRNA*^*Gly*^ (1 bp), and *16S rRNA* (1 bp). Additionally, the *D*. *extinctorium* mitogenome accommodates 1393 bp of intergenic spacers distributed across 28 regions, ranging in size from 3 to 648 bp (Table 2).

Regarding nucleotide composition, the *D*. *extinctorium* mitogenome is comprised of A (27.73%), T (42.47%), G (18.08%), and C (11.71%), demonstrating a conspicuous AT bias. These findings parallel not only those observed in numerous mollusks [60,61] but also in certain crustaceans like crabs and lobsters [62,63]. The cumulative A + T (%) content of the mitogenomes stands at 70.20%. Calculated for the selected complete mitogenomes, the AT-skew of the *D*. *extinctorium* mitogenome is negative (−0.210), while the GC-skew is positive (0.214), implying a higher abundance of Ts and Cs than As and Gs. These outcomes align with those identified in specific Neritidae species [57].

### Transfer RNAs, ribosomal RNAs

**Fig 2:**
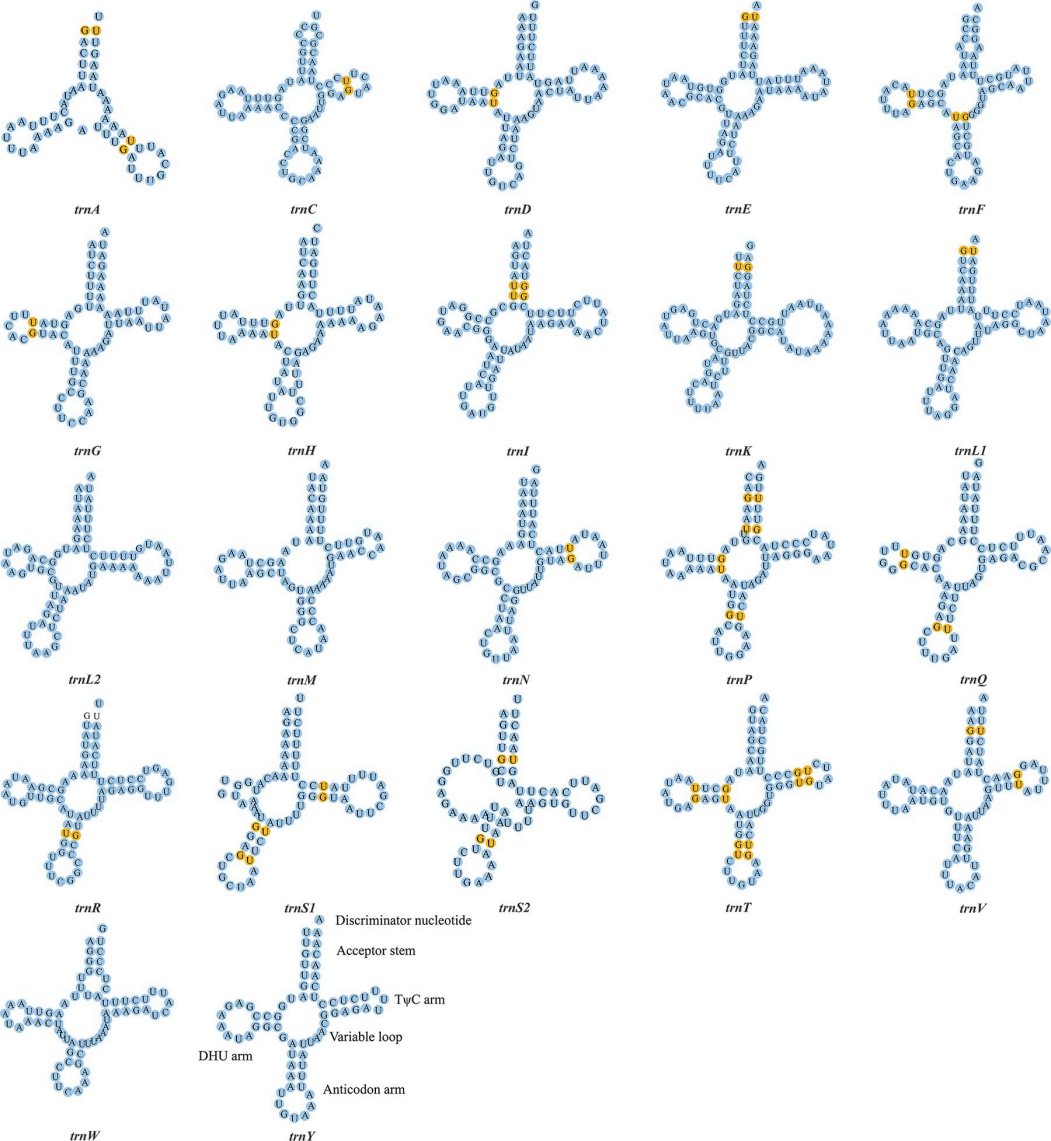
Secondary structure of the tRNA genes in the mitogenome of *D*. *extinctorium*. The tRNAs are labeled with the abbreviations of their corresponding amino acids. Blue dots indicate normal conditions and yellow dots indicate base mismatches.

Similar to the prevailing pattern in many invertebrate species [64,65], the mitogenome of *D*. *extinctorium* harbors a total of 22 tRNA genes. Among these, fourteen are encoded by the heavy strand (H-), while the remaining ones are encoded by the light strand (L-). Across the entire mitogenome, the size of tRNA molecules spans from 51 to 75 bp, collectively encompassing a length of 1485 bp, characterized by a pronounced AT bias (70.23%). The AT-skew and GC-skew values are recorded as– 0.014 and 0.158, respectively, signifying a subtle inclination towards adenine usage and a conspicuous predilection for guanine usage (Table 3). The *tRNA*^*Ser1*^ and *tRNA*^*Ser2*^, due to the absence of dihydrouracil (DHU) arms, along with *tRNA*^*Ala*^, due to the lack of a TΨC arm, are unable to adopt a secondary structure. Conversely, other tRNAs possess the capacity to fold into a conventional clover-leaf secondary structure. Notably, the structural variation observed in *tRNA*^*Ser1*^ corresponds with the *tRNA*^*Ser1*^ configuration documented in other invertebrate mitogenomes [66]. Moreover, G-C mismatches are evident in all tRNAs except *tRNA*^*Leu2*^, *tRNA*^*Met*^, *tRNA*^*Trp*^, and *tRNA*^*Tyr*^. (Fig 2. Secondary structure of the tRNA genes in the mitogenome of *D*. *extinctorium*. The tRNAs are labeled with the abbreviations of their corresponding amino acids. Blue dots indicate normal conditions and yellow dots indicate base mismatches.).

**Table 3:** Nucleotide contents of the coding and non-coding regions of the mitochondrial genome of *D*. *extinctorium*, indicating AT-, GC-skew ratios.

Region	Size(bp)	A (%)	T (%)	G (%)	C (%)	A+T (%)	AT-skew	GC-skew
Mitogenome	16608	27.73	42.47	18.08	11.72	70.20	-0.210	0.213
*COX1*	1551	23.92	43.13	20.05	12.89	67.05	-0.287	0.217
*COX2*	693	27.13	38.96	20.49	13.42	66.09	-0.179	0.209
*ATP8*	159	27.67	44.65	15.72	11.95	72.32	-0.235	0.136
*ATP6*	696	23.13	46.70	16.67	13.51	69.83	-0.337	0.105
*COX3*	780	21.67	42.31	22.31	13.72	63.98	-0.323	0.238
*NAD3*	357	19.89	47.90	20.73	11.48	67.79	-0.413	0.287
*NAD1*	945	24.02	45.29	18.20	12.49	69.31	-0.307	0.186
*NAD5*	1872	26.82	42.63	16.61	13.94	69.45	-0.228	0.087
*NAD4*	1305	25.21	46.13	16.86	11.80	71.34	-0.293	0.176
*NAD4l*	303	26.73	43.89	19.80	9.57	70.62	-0.243	0.348
*NAD6*	504	26.39	46.43	18.65	8.53	72.82	-0.275	0.372
*Cytb*	1140	24.39	44.39	17.81	13.42	68.78	-0.291	0.140
*NAD2*	1089	25.34	46.37	18.64	9.64	71.71	-0.293	0.318
*tRNAs*	1485	34.61	35.62	17.24	12.53	70.23	-0.014	0.158
*rRNAs*	2260	34.91	38.14	17.08	9.87	73.05	-0.044	0.268
*PCGs*	11394	24.84	44.25	18.47	12.44	69.09	-0.281	0.195

The sizes of the *12S rRNA* and *16S rRNA* components are 896 bp and 1364 bp, respectively, typically demarcated by *tRNA*^*Val*^ (Table 2). These dimensions align comparably with those observed in other invertebrate species. The A-T content of the rRNAs is determined to be 73.05%. AT-skew and GC-skew values are recorded as– 0.044 and 0.268, respectively, indicating a modest tendency towards adenine utilization and a marked preference for guanine utilization (Table 3). The control region (CR), positioned between *tRNA*^*Thr*^ and *tRNA*^*Phe*^, spans a length of 648 bp.

### PCGs and codon usage

**Fig 3:**
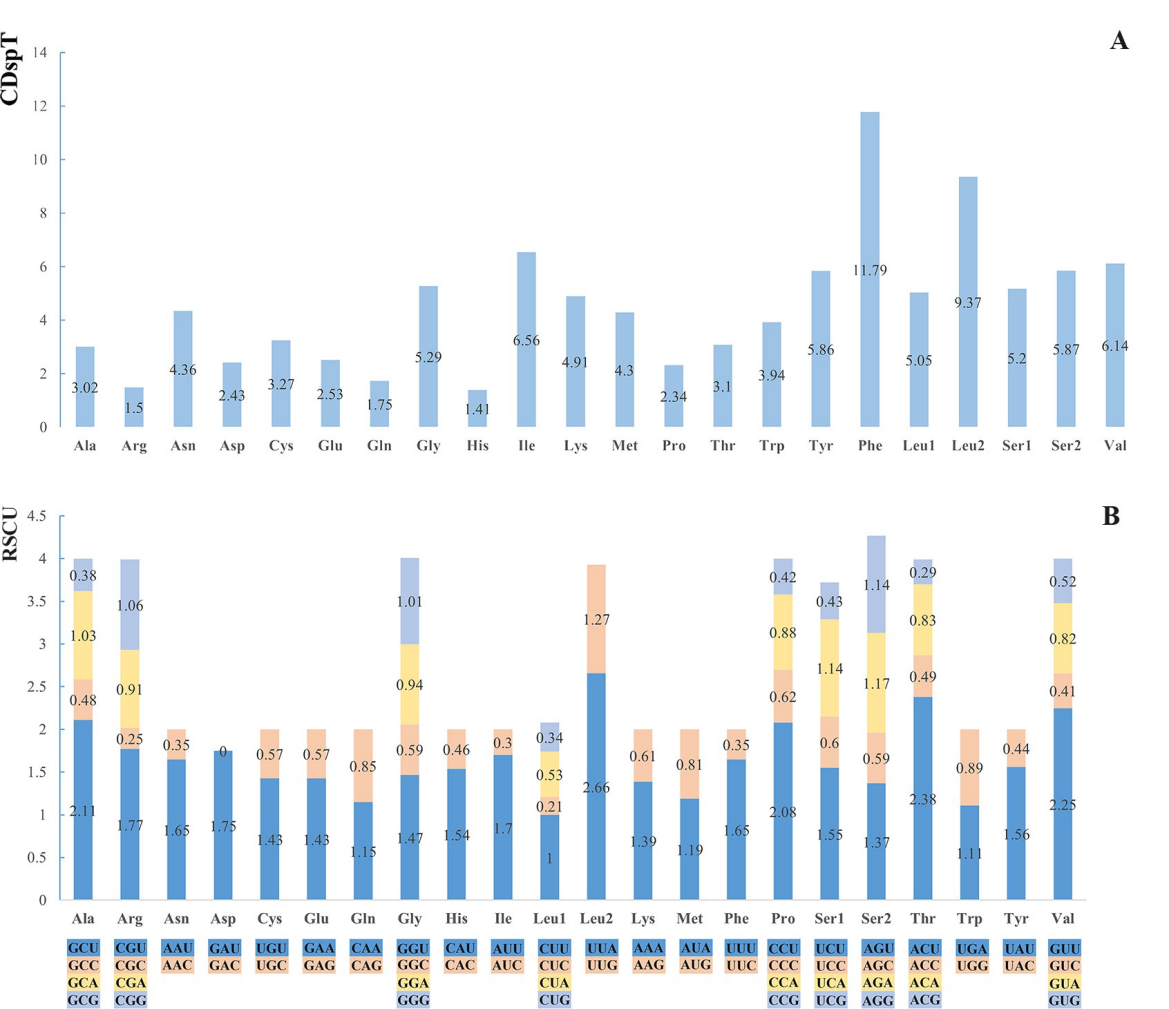
Codon usage patterns in the mitogenome of *D*. *extinctorium*. CDspT, codons per thousand codons. Codon families are provided on the x-axis (A) and the relative synonymous codon usage (RSCU) (B).

The result presents the initiation and termination codons for all Protein-Coding Genes (PCGs) within *D*. *extinctorium* in Table 3. The mitochondrial genome of D. extinctorium encompasses a total of 13 PCGs, comprising a cytochrome b (*Cytb*), two ATPases (*ATP6* and *ATP8*), three cytochrome oxidases (*COI–III*), and seven NADH dehydrogenases (*ND1–6* and *ND4L*). This configuration aligns with the established structural pattern observed in the Muricidae family [64]. The collective length of these 13 PCGs amounts to 11,484 bp. Within this set, the individual PCGs exhibit a range of lengths spanning from 159 to 1,872 bp. Notably, the average A+T content stands at 69.13%, with variations across the spectrum from 63.98% (*COIII*) to 72.82% (*ND6*) (Table 2). The AT-skew and GC-skew values are calculated as -0.281 and 0.195, respectively (Table 4). It is noteworthy that all PCGs commence with the initiation codon ATG, except for *ND4*, which employs ATT as its start codon. Furthermore, the majority of PCGs terminate with TAA, whereas *ND4L*, *ND5*, and *ND5* employ TAG as their respective stop codons (Table 4). Examining the amino acid utilization in *D*. *extinctorium*, *tRNA*^*Phe*^ emerges as the most frequently employed, while *tRNA*^*His*^ is the least prevalent (Fig 2). Relative synonymous codon usage (RSCU) values for the 13 PCGs in *D*. *extinctorium* are presented in Table 4 and Fig 3. Among these, UUA (Leu) ranks as the most frequently encountered codon, whereas CUC (Leu) stands as the least common codon. (Fig 3. Codon usage patterns in the mitogenome of *D*. *extinctorium*. CDspT, codons per thousand codons. Codon families are provided on the x-axis (A) and the relative synonymous codon usage (RSCU) (B)).

**Table 4:** Relative synonymous codon usage (RSCU) in the mitogenomes of *D*. *extinctorium*.

Codon	Count	RSCU	Codon	Count	Codon	Codon	Count	RSCU	Codon	Count	Codon
GCU(A)	84.0	2.11	CCU(P)	64.0	2.08	AGA(S)	85.0	1.17	CAU(H)	57.0	1.54
GCC(A)	19.0	0.48	CCC(P)	19.0	0.62	AGG(S)	83.0	1.14	CAC(H)	17.0	0.46
GCA(A)	41.0	1.03	CCA(P)	27.0	0.88	AUU(I)	293.0	1.70	ACU(T)	97.0	2.38
GCG(A)	15.0	0.38	CCG(P)	13.0	0.42	AUC(I)	52.0	0.30	ACC(T)	20.0	0.49
UGU(C)	123.0	1.43	CAA(Q)	53.0	1.15	AAA(K)	179.0	1.39	ACA(T)	34.0	0.83
UGC(C)	49.0	0.57	CAG(Q)	39.0	0.85	AAG(K)	79.0	0.61	ACG(T)	12.0	0.29
GAU(D)	112.0	1.75	CGU(R)	35.0	1.77	UUA(L)	336.0	2.66	GUU(V)	182.0	2.25
GAC(D)	16.0	0.25	CGC(R)	5.0	0.25	UUG(L)	160.0	1.27	GUC(V)	33.0	0.41
GAA(E)	95.0	1.43	CGA(R)	18.0	0.91	CUU(L)	126.0	1.00	GUA(V)	66.0	0.82
GAG(E)	38.0	0.57	CGG(R)	21.0	1.06	CUC(L)	26.0	0.21	GUG(V)	42.0	0.52
UUU(F)	512.0	1.65	UCU(S)	113.0	1.55	CUA(L)	67.0	0.53	UGA(W)	115.0	1.11
UUC(F)	108.0	0.35	UCC(S)	44.0	0.60	CUG(L)	43.0	0.34	UGG(W)	92.0	0.89
GGU(G)	102.0	1.47	UCA(S)	83.0	1.14	AUA(M)	135.0	1.19	UAU(Y)	240.0	1.56
GGC(G)	41.0	0.59	UCG(S)	31.0	0.43	AUG(M)	91.0	0.81	UAC(Y)	68.0	0.44
GGA(G)	65.0	0.94	AGU(S)	100.0	1.37	AAU(N)	189.0	1.65	UAA(*)	174.0	1.25
GGG(G)	70.0	1.01	AGC(S)	43.0	0.59	AAC(N)	40.0	0.35	UAG(*)	105.0	0.75

### Gene re-arrangement

**Fig 4:**
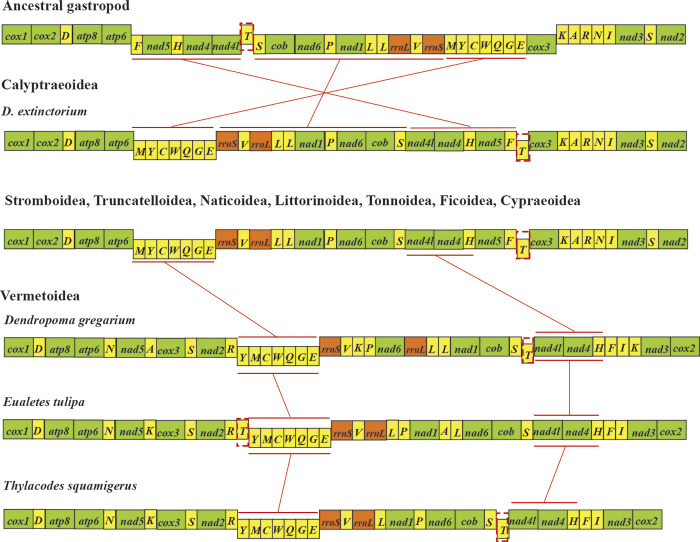
Comparison of mitochondrial gene rearrangements of the *D*. *extinctorium*. The green squares represent PCGs, the yellow squares represent tRNAs, and the orange squares represent rRNAs. The position at the top indicates that it is encoded in the H chain, and the position at the bottom indicates that it is encoded in the L chain.

Rearrangements in mitochondrial gene order present an autonomous dataset for resolving evolutionary relationships. Shared patterns of mitogenome gene order rearrangements among distinct taxonomic groups are likely indicative of common ancestry rather than products of convergent evolution [6,67]. In comparison to the ancestral gastropod gene arrangement, significant rearrangements are evident in the mitogenome of *D*. *extinctorium*. As illustrated in Fig 4, a minimum of three gene clusters (or genes) differ notably from the conventional arrangement, encompassing 15 tRNA genes (*M*, *Y*, *C*, *W*, *Q*, *G*, *E*, *V*, *L*, *P*, *S*, *H*, *F*, and *T*), as well as eight protein-coding genes (*16S rRNA*, *12S rRNA*, *NAD1*, *NAD6*, *Cytb*, *NAD4L*, *NAD4*, and *NAD5*). The rearrangement of these three gene clusters (or genes) is detailed as follows (Fig 4): (1) The *M-Y-C-W-Q-G-E* cluster has relocated downstream of *ATP6*; (2) The *T* cluster has shifted downstream of *F*; (3) The *F-ND5-H-ND4-ND4L-S-Cytb-ND6-P-ND1-L-16S-V-12S* underwent inversion and translocation. (Fig 4. Comparison of mitochondrial gene rearrangements of the *D*. *extinctorium*. The green squares represent PCGs, the yellow squares represent tRNAs, and the orange squares represent rRNAs. The position at the top indicates that it is encoded in the H chain, and the position at the bottom indicates that it is encoded in the L chain.)

Furthermore, mitochondrial gene rearrangements have frequently been linked to heightened rates of evolution [68]. Prior investigations have identified a notable positive correlation in mitochondrial genomes between rates of gene order rearrangement and accelerated evolutionary rates [69]. Intriguingly, when compared to the extensive gene rearrangements observed in Lottiidae, Littorinimorpha exhibits minimal differences in genetic order, with the exception of Vermetoidea [70]. We postulate that this circumstance could be attributed to the relatively modest variations in genome size among Littorinimorpha species, ranging from 15,127 bp to 17,195 bp (Tab 1), while the mitochondrial genome size within Lottiidae spans from 16,319 bp to 26,835 bp. Further investigations are warranted to scrutinize this association within a broader spectrum of Gastropoda groups.

In the context of gene rearrangement patterns, three primary categories are recognized [71]: (1) shuffling, where genes migrate from their original locations to adjacent positions on the same strand, typically without traversing protein-coding genes; (2) translocation, in which genes traverse several genes, often including protein-coding genes, relocating from their original positions to new sites; (3) inversion, involving the switch of genes from one strand to the other. Based on the characteristics of mitochondrial sequences, our analysis suggests that inversion and translocation are the predominant types of rearrangements observed in *D*. *extinctorium*.

Furthermore, we conducted a comparative examination of the gene order in *D*. *extinctorium* against other superfamilies within Littorinimorpha. With the exception of Vermetoidea, the gene order across other superfamilies remains largely consistent. Notably, in Vermetoidea, significant deviations in gene order primarily pertain to tRNAs. In addition, the *M-Y-C-W-Q-G-E* cluster within the mitochondrial genome of Vermetoidea has undergone inversion, a phenomenon observed in other gastropod mitochondrial genomes [72], resulting in disruption and rearrangement. Intriguingly, a remarkably similar set of genes undergoes rearrangement in the common ancestor of Caenogastropoda, although the integrity of the *M-Y-C-W-Q-G-E* cluster is maintained [22,73,74]. These findings align with the conclusions drawn from gene order-based phylogenetic analysis, underscoring the utility of comparing mitochondrial gene rearrangements as a valuable tool in phylogenetic studies.

### Phylogenetic relationships

**Fig 5:**
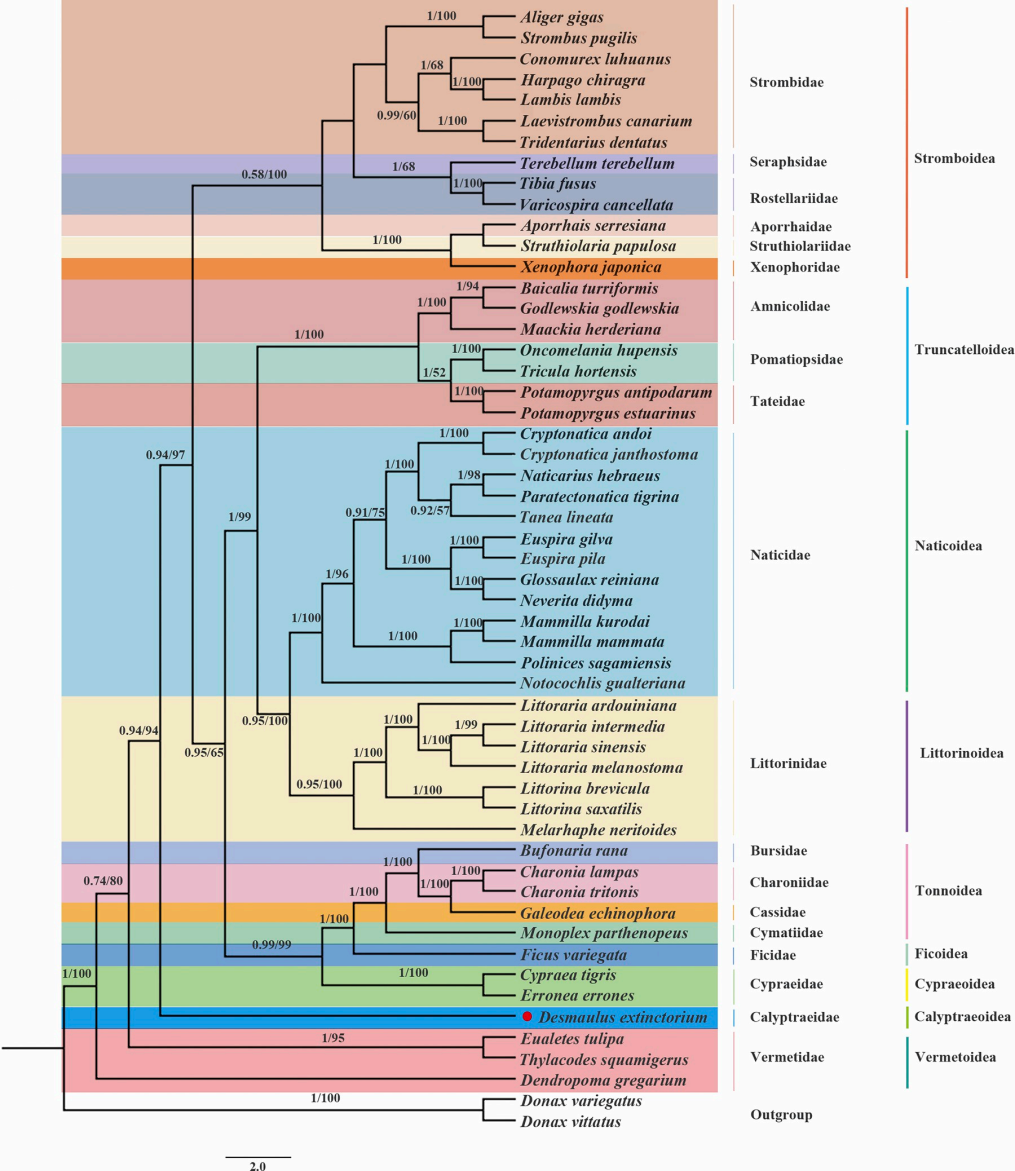
The phylogenetic tree was inferred from the nucleotide sequences of 13 mitogenome PCGs using BI and ML methods. Numbers on branches indicate posterior probability (BI) and bootstrap support (ML).

In this current study, we conducted an analysis of phylogenetic relationships using the sequences of 13 protein-coding genes (PCGs). The primary objective was to gain insights into the interrelationships within the Littorinimorpha clade, focusing on *D*. *extinctorium*. Additionally, we included 51 other well-known Littorinimorpha species in our analysis, with *Donax variegatus* and *Donax vittatus* serving as outgroups. Both the Maximum Likelihood (ML) tree and the Bayesian Inference (BI) tree revealed consistent topological structures, although they exhibited varying degrees of support values. Notably, BI generally yielded higher support values, with most nodes having a support value of 1. In contrast, the support values in ML, except for three nodes in the Stromboidea superfamily, were below 70, and the majority of other branches had support values above 90. Consequently, we present and display only one topology (ML) with both support values. (Fig 5. The phylogenetic tree was inferred from the nucleotide sequences of 13 mitogenome PCGs using BI and ML methods. Numbers on branches indicate posterior probability (BI) and bootstrap support (ML)).

Among the 19 families encompassed within our phylogenetic tree, each individual family constitutes a monophyletic clade, bolstered by elevated nodal support values. Phylogenetic analysis showed that nine superfamilies within the Littorinimorpha show the following relationship: ((((((Naticoidea + Littorinoidea) + Truncatelloidea) + Tonnoidea + Ficoidea + Cypraeoidea) +Stromboidea) + Calyptraeoidea) + Vermetoidea), and all nine of them are monophyletic groups, some previous studies have shown that this is plausible [75,76], and Naticoidea and Littorinoidea are the closest sisters to each other. Additionally, phylogenetic tree showed that (Tonnoidea + Ficoidea + Cypraeoidea) formed a clade which showing were sister groups in this tree, while *D*. *extinctoriu*m alone forms a Calyptraeoidea clade, and (Calyptraeoidea + (Stromboidea + (Tonnoidea + Ficoidea + Cypraeoidea) + Truncatelloidea + (Naticoidea + Littorinoidea))) formed a clade. Vermetoidea is placed at the basal position of the monophyletic Littorinimorpha, this is consistent with previous research [73], and this can also be related to the results of gene re-arrangements, only the Vermetoidea has a significantly different genetic sequence from the rest of the species, so Vermetoidea is at the bottom of the phylogenetic tree. Stromboidea is the superfamily containing the largest number of families, it is a highly diverse group. Stromboidea is currently understood to comprise six extant families: Aporrhaidae, Rostellariidae, Seraphsidae, Strombidae, Struthiolariidae and Xenophoridae. Within each superfamily, each family forms a distinct clade. The results of phylogenetic relationships in the superfamil were consistent with the findings of Irwin et al [38]. In our study, Naticidae is the family of which most species have been included, representing a large number of genera. The Littorinidae are its sister group, of which a substantial number of species has been included in our study, however only representing a selection of the genera.

## Conclusion

In this investigation, we conducted the sequencing of the mitogenome of *D*. *extinctorium* employing next-generation sequencing techniques, thus yielding novel mitochondrial data pertinent to Calyptraeidae. An exhaustive examination of the mitogenome of *D*. *extinctorium* revealed its substantial resemblance to other representatives of the Littorinimorpha order, characterized by several notable features, including AT-skew and a codon usage bias, among others. Comparative analysis with the ancestral gastropod indicated a noteworthy rearrangement in the gene order of the *D*. *extinctorium* mitogenome. The Littorinimorpha exhibited four distinct rearrangement patterns, with their rearrangement similarity consistently mirroring their phylogenetic relationships. Our phylogenetic tree displayed both congruities and disparities when compared to preceding studies. Phylogenetic analyses indicated the formation of an exclusive Calyptraeoidea clade by *D*. *extinctorium*, whereas (Calyptraeoidea + (Stromboidea + (Tonnoidea + Ficoidea + Cypraeoidea) + Truncatelloidea + (Naticoidea + Littorinoidea))) constituted a distinct clade. Despite a limited number of species available for a robust phylogenetic analysis, our phylogeny garnered statistical support and aspires to provide a rational framework for future phylogenetic inquiries within the realm of Calyptraeoidea. These findings not only offer insights into the gene arrangement characteristics within Littorinimorpha mitogenomes but also establish the groundwork for further explorations into the phylogenetic aspects of Littorinimorpha.

## References

[1] Ballard J, Whitlock M. Ballard JWO, Whitlock MC. The incomplete natural history of mitochondria. Mol Ecol 13: 729–744. Molecular ecology. 2004;13:729–44. doi: 10.1046/j.1365-294X.2003.02063.x 15012752

[2] Gissi C, Iannelli F, Pesole G. Evolution of the mitochondrial genome of Metazoa as exemplified by comparison of congeneric species. Heredity. 2008;101(4):301–20. doi: 10.1038/hdy.2008.62 18612321

[3] Kurabayashi A, Sumida M, Yonekawa H, Glaw F, Vences M, Hasegawa M. Phylogeny, Recombination, and Mechanisms of Stepwise Mitochondrial Genome Reorganization in Mantellid Frogs from Madagascar. Molecular Biology and Evolution. 2008;25(5):874–91. doi: 10.1093/molbev/msn031 18263605

[4] Saccone C, De Giorgi C, Gissi C, Pesole G, Reyes A. Evolutionary genomics in Metazoa: the mitochondrial DNA as a model system. Gene. 1999;238(1):195–209. doi: 10.1016/s0378-1119(99)00270-x 10570997

[5] Ye YY, Wu CW, Li JJ. Genetic Population Structure of Macridiscus multifarius (Mollusca: Bivalvia) on the Basis of Mitochondrial Markers: Strong Population Structure in a Species with a Short Planktonic Larval Stage. PLOS ONE. 2016;10(12):e0146260. doi: 10.1371/journal.pone.0146260 26720602 PMC4697803

[6] Zhang Y, Gong L, Lu X, Jiang L, Liu B, Liu L, et al. Gene rearrangements in the mitochondrial genome of Chiromantes eulimene (Brachyura: Sesarmidae) and phylogenetic implications for Brachyura. International Journal of Biological Macromolecules. 2020;162:704–14. 10.1016/j.ijbiomac.2020.06.196. 32590088

[7] Kumar V, Tyagi K, Chakraborty R, Prasad P, Kundu S, Tyagi I, et al. The Complete Mitochondrial Genome of endemic giant tarantula, Lyrognathus crotalus (Araneae: Theraphosidae) and comparative analysis. Scientific Reports. 2020;10(1):74. doi: 10.1038/s41598-019-57065-8 31919395 PMC6952441

[8] Ruan H, Li M, Li Z, Huang J, Chen W, Sun J, et al. Comparative Analysis of Complete Mitochondrial Genomes of Three Gerres Fishes (Perciformes: Gerreidae) and Primary Exploration of Their Evolution History. International Journal of Molecular Sciences [Internet]. 2020; 21(5). doi: 10.3390/ijms21051874 32182936 PMC7084342

[9] Yang Y, Li Q, Kong L, Yu H. Mitogenomic phylogeny of Nassarius (Gastropoda: Neogastropoda). Zoologica Scripta. 2019.

[10] Yang Y, Liu H, Qi L, Kong L, Li Q. Complete Mitochondrial Genomes of Two Toxin-Accumulated Nassariids (Neogastropoda: Nassariidae: Nassarius) and Their Implication for Phylogeny. International Journal of Molecular Sciences [Internet]. 2020; 21(10). doi: 10.3390/ijms21103545 32429583 PMC7278921

[11] Lee SY, Lee HJ, Kim YK. Comparative analysis of complete mitochondrial genomes with Cerithioidea and molecular phylogeny of the freshwater snail, Semisulcospira gottschei (Caenogastropoda, Cerithioidea). International Journal of Biological Macromolecules. 2019;135:1193–201. doi: 10.1016/j.ijbiomac.2019.06.036 31176862

[12] Knudsen J. OBSERVATIONS ON CALYPTRAEA EXTINCTORIUM LAMARCK, 1822 (PROSOBRANCHIA: CALYPTRAEIDAE) FROM HONG KONG. In: Morton B, editor. The Marine Flora and Fauna of Hong Kong and Southern China IV: Hong Kong University Press; 1997. p. 371–80.

[13] Teso V, Penchaszadeh PE. Development of the gastropod Trochita pileus (Calyptraeidae) in the sub-Antarctic Southwestern Atlantic. Polar biology. 2019;42(1):171–8.

[14] Holtheuer J, Aldea C, Schories D, Gallardo C. The natural history of Calyptraea aurita (Reeve, 1859) from Southern Chile (Gastropoda, Calyptraeidae). ZooKeys. 2018;798:1–22. 10.3897/zookeys.798.25736 PMC6262045 30532280

[15] Cledón M, Nuñez JD, Ocampo EH, Sigwart JD. Sexual traits plasticity of the potentially invasive limpet Bostrycapulus odites (Gastropoda: Calyptraeidae) within its natural distribution in South America. Marine Ecology. 2016;37(2):433–41. doi: 10.1111/maec.12329

[16] Collin R. Development, phylogeny, and taxonomy of Bostrycapulus (Caenogastropoda: Calyptraeidae), an ancient cryptic radiation. Zoological Journal of the Linnean Society. 2005;144(1):75–101. doi: 10.1111/j.1096-3642.2005.00162.x

[17] Cunha RL, Grande C, Zardoya R. Neogastropod phylogenetic relationships based on entire mitochondrial genomes. BMC Evol Biol. 2009;9:210. Epub 2009/08/25. doi: 10.1186/1471-2148-9-210 ; PubMed Central PMCID: PMC2741453. 19698157 PMC2741453

[18] Collin R. The effects of mode of development on phylogeography and population structure of North Atlantic Crepidula (Gastropoda: Calyptraeidae). Molecular Ecology. 2010;10(9):2249–62. 10.1046/j.1365-294x.2001.01372.x 11555267

[19] Bouchet P, Rocroi JP, Fryda J, Hausdorf B, Ponder W, Valdes A, et al. CLASSIFICATION AND NOMENCLATOR OF GASTROPOD FAMILIES. Malacologia. 2005;47(1/2):p.1–397.

[20] Colgan DJ, Ponder WF, Beacham E, Macaranas J. Molecular phylogenetics of Caenogastropoda (Gastropoda: Mollusca). Molecular Phylogenetics and Evolution. 2007;42(3):717–37. doi: 10.1016/j.ympev.2006.10.009 17127080

[21] Osca D, Templado J, Zardoya R. Caenogastropod mitogenomics. Mol Phylogenet Evol. 2015;93:118–28. Epub 2015/07/30. doi: 10.1016/j.ympev.2015.07.011 . 26220836

[22] Cunha RL, Grande C, Zardoya R. Neogastropod phylogenetic relationships based on entire mitochondrial genomes. BMC Evolutionary Biology. 2009;9(1):210. doi: 10.1186/1471-2148-9-210 19698157 PMC2741453

[23] Zhao Q-P, Zhang SH, Deng Z-R, Jiang M-S, Nie P. Conservation and variation in mitochondrial genomes of gastropods Oncomelania hupensis and Tricula hortensis, intermediate host snails of Schistosoma in China. Molecular Phylogenetics and Evolution. 2010;57(1):215–26. doi: 10.1016/j.ympev.2010.05.026 20595013

[24] Riedel F. Recognition of the superfamily Ficoidea Meek 1864 and definition of the Thalassocynidae fam. nov. (Gastropoda). 1994.

[25] Wang Q, Liu H, Yue C, Xie X, Li D, Liang M, et al. Characterization of the complete mitochondrial genome of Ficus variegata (Littorinimorpha: Ficidae) and molecular phylogeny of Caenogastropoda. Mitochondrial DNA Part B. 2021;6(3):1126–8. doi: 10.1080/23802359.2021.1901628 33796763 PMC7995913

[26] Jiang D, Zheng X, Zeng X, Kong L, Li Q. The complete mitochondrial genome of Harpago chiragra and Lambis lambis (Gastropoda: Stromboidea): implications on the Littorinimorpha phylogeny. Scientific Reports. 2019;9(1):17683. doi: 10.1038/s41598-019-54141-x 31776396 PMC6881320

[27] Aljanabi SM, Martinez I. Universal and rapid salt-extraction of high quality genomic DNA for PCR-based techniques. Nucleic Acids Research. 1997;25(22):4692–3. doi: 10.1093/nar/25.22.4692 9358185 PMC147078

[28] Dierckxsens N, Mardulyn P, Smits G. NOVOPlasty: de novo assembly of organelle genomes from whole genome data. Nucleic Acids Research. 2017;45(4):e18-e. doi: 10.1093/nar/gkw955 28204566 PMC5389512

[29] Bernt M, Donath A, Jühling F, Externbrink F, Florentz C, Fritzsch G, et al. MITOS: Improved de novo metazoan mitochondrial genome annotation. Molecular Phylogenetics and Evolution. 2013;69(2):313–9. doi: 10.1016/j.ympev.2012.08.023 22982435

[30] Altschul SF, Madden TL, Schäffer AA, Zhang J, Zhang Z, Miller W, et al. Gapped BLAST and PSI-BLAST: a new generation of protein database search programs. Nucleic Acids Research. 1997;25(17):3389–402. doi: 10.1093/nar/25.17.3389 9254694 PMC146917

[31] Grant JR, Stothard P. The CGView Server: a comparative genomics tool for circular genomes. Nucleic Acids Research. 2008;36(suppl_2):W181–W4. doi: 10.1093/nar/gkn179 18411202 PMC2447734

[32] Kumar S, Stecher G, Tamura K. MEGA7: Molecular Evolutionary Genetics Analysis Version 7.0 for Bigger Datasets. Molecular Biology and Evolution. 2016;33(7):1870–4. doi: 10.1093/molbev/msw054 27004904 PMC8210823

[33] Xia X, Xie Z. DAMBE: Software Package for Data Analysis in Molecular Biology and Evolution. Journal of Heredity. 2001;92(4):371–3. doi: 10.1093/jhered/92.4.371 11535656

[34] Machkour-M’Rabet S, Hanes MM, Martínez-Noguez JJ, Cruz-Medina J, García-De León FJ. The queen conch mitogenome: intra- and interspecific mitogenomic variability in Strombidae and phylogenetic considerations within the Hypsogastropoda. Scientific Reports. 2021;11(1):11972. doi: 10.1038/s41598-021-91224-0 34099752 PMC8184947

[35] Zhao Z-y Tu Z-g, Bai L-r, Cui J. Characterization of an endangered marine strombid gastropod Strombus luhuanus complete mitochondrial genome. Conservation Genetics Resources. 2018;10(1):55–7. doi: 10.1007/s12686-017-0764-7

[36] Jiang D, Zheng X, Zeng X, Kong L, Li Q. The complete mitochondrial genome of Harpago chiragra and Lambis lambis (Gastropoda: Stromboidea): implications on the Littorinimorpha phylogeny. Sci Rep. 2019;9(1):17683. Epub 2019/11/30. doi: 10.1038/s41598-019-54141-x ; PubMed Central PMCID: PMC6881320. 31776396 PMC6881320

[37] Lee HT, Liao CH, Huang CW, Chang YC, Hsu TH. The complete mitochondrial genome of Laevistrombus canarium (Gastropoda: Stromboidae). Mitochondrial DNA B Resour. 2021;6(2):591–2. Epub 2021/02/26. doi: 10.1080/23802359.2021.1875920 ; PubMed Central PMCID: PMC7889242. 33628941 PMC7889242

[38] Irwin AR, Strong EE, Kano Y, Harper EM, Williams ST. Eight new mitogenomes clarify the phylogenetic relationships of Stromboidea within the caenogastropod phylogenetic framework. Molecular Phylogenetics and Evolution. 2021;158:107081. doi: 10.1016/j.ympev.2021.107081 33482382

[39] Peretolchina TE, Sitnikova TY, Sherbakov DY. The complete mitochondrial genomes of four Baikal molluscs from the endemic family Baicaliidae (Caenogastropoda: Truncatelloida). Journal of Molluscan Studies. 2020;86(3):201–9. doi: 10.1093/mollus/eyaa004

[40] Sharbrough J, Bankers L, Cook E, Fields PD, Jalinsky J, McElroy KE, et al. Single-molecule Sequencing of an Animal Mitochondrial Genome Reveals Chloroplast-like Architecture and Repeat-mediated Recombination. Mol Biol Evol. 2023;40(1). Epub 2023/01/11. doi: 10.1093/molbev/msad007 ; PubMed Central PMCID: PMC9874032. 36625177 PMC9874032

[41] Zhong S, Liu Y, Huang G, Huang L. The first complete mitochondrial genome of Bursidae from Bufonaria rana (Caenogastropoda: Tonnoidea). Mitochondrial DNA Part B. 2020;5(3):2585–6. doi: 10.1080/23802359.2020.1781575

[42] Liu H, Yang Y, Sun Se, Kong L, Li Q. Mitogenomic phylogeny of the Naticidae (Gastropoda: Littorinimorpha) reveals monophyly of the Polinicinae. Zoologica Scripta. 2020;49(3):295–306. doi: 10.1111/zsc.12412

[43] Li PY, Yang Y, Li YG, Sun SE. The complete mitochondrial genome of Glossaulax reiniana (Littorinimorpha: Naticidae). Mitochondrial DNA B Resour. 2018;3(2):1263–4. Epub 2018/10/26. doi: 10.1080/23802359.2018.1532829 ; PubMed Central PMCID: PMC7800891. 33474486 PMC7800891

[44] Pu L, Liu H, Yang M, Li B, Xia G, Shen M, et al. Complete mitochondrial genome of tiger cowrie Cypraea tigris (Linnaeus, 1758). Mitochondrial DNA B Resour. 2019;4(2):2755–6. Epub 2019/07/26. doi: 10.1080/23802359.2019.1627933 ; PubMed Central PMCID: PMC7687430. 33365715 PMC7687430

[45] Rawlings TA, MacInnis MJ, Bieler R, Boore JL, Collins TM. Sessile snails, dynamic genomes: gene rearrangements within the mitochondrial genome of a family of caenogastropod molluscs. BMC Genomics. 2010;11:440. Epub 2010/07/21. doi: 10.1186/1471-2164-11-440 ; PubMed Central PMCID: PMC3091637. 20642828 PMC3091637

[46] Li M-Y, Li Y-L, Xing T-F, Liu J-X. First mitochondrial genome of a periwinkle from the genus Littoraria: Littoraria sinensis. Mitochondrial DNA Part B. 2019;4(2):4124–5. doi: 10.1080/23802359.2019.1692718 33366348 PMC7707726

[47] Bai J, Guo Y, Feng J, Ye Y, Li J, Yan C, et al. The complete mitochondrial genome and phylogenetic analysis of Littorina brevicula (Gastropoda, Littorinidea). Mitochondrial DNA B Resour. 2020;5(3):2280–1. Epub 2020/12/29. doi: 10.1080/23802359.2020.1772145 ; PubMed Central PMCID: PMC7510672. 33367008 PMC7510672

[48] Fourdrilis S, de Frias Martins AM, Backeljau T. Relation between mitochondrial DNA hyperdiversity, mutation rate and mitochondrial genome evolution in Melarhaphe neritoides (Gastropoda: Littorinidae) and other Caenogastropoda. Sci Rep. 2018;8(1):17964. Epub 2018/12/21. doi: 10.1038/s41598-018-36428-7 ; PubMed Central PMCID: PMC6299273. 30568252 PMC6299273

[49] Fernández-Pérez J, Nantón A, Ruiz-Ruano FJ, Camacho JPM, Méndez J. First complete female mitochondrial genome in four bivalve species genus Donax and their phylogenetic relationships within the Veneroida order. PLoS One. 2017;12(9):e0184464. Epub 2017/09/09. doi: 10.1371/journal.pone.0184464 ; PubMed Central PMCID: PMC5590976. 28886105 PMC5590976

[50] Perna NT, Kocher TD. Patterns of nucleotide composition at fourfold degenerate sites of animal mitochondrial genomes. Journal of Molecular Evolution. 1995;41(3):353–8. doi: 10.1007/BF00186547 7563121

[51] Huelsenbeck JP, Ronquist F. MRBAYES: Bayesian inference of phylogenetic trees. Bioinformatics. 2001. doi: 10.1093/bioinformatics/17.8.754 11524383

[52] Lam-Tung N, Schmidt HA, Arndt VH, Quang MB. IQ-TREE: A Fast and Effective Stochastic Algorithm for Estimating Maximum-Likelihood Phylogenies. Molecular Biology & Evolution. 2015;(1):268–74. 10.1093/molbev/msu300 PMC4271533 25371430

[53] Liu L, Teslenko M, Hohna S, Ayres DL, Paul VDM, Darling A, et al. MrBayes 3.2: Efficient Bayesian Phylogenetic Inference and Model Choice Across a Large Model Space. Systematic Biology. 2012;61(3):539–42. doi: 10.1093/sysbio/sys029 22357727 PMC3329765

[54] Nylander JAA, Ronquist F, Huelsenbeck JP, Nievesaldrey JL. doi: 10.1080/10635150490264699 Bayesian Phylogenetic Analysis of Combined Data. 2013. 14965900

[55] Posada D, Crandall KA. MODELTEST: testing the model of DNA substitution. Bioinformatics. 1998;14(9):817–8. doi: 10.1093/bioinformatics/14.9.817 9918953

[56] Rambaut A. FigTree, version 1.4.3 2018 [cited 2016 1 July]. Available from: http://tree.bio.ed.ac.uk/software/figtree/.

[57] Miao J, Feng J, Liu X, Yan C, Ye Y, Li J, et al. Sequence comparison of the mitochondrial genomes of five brackish water species of the family Neritidae: Phylogenetic implications and divergence time estimation. Ecology and Evolution. 2022;12(6):e8984. doi: 10.1002/ece3.8984 35784089 PMC9170520

[58] Xu M, Gu Z, Huang J, Guo B, Jiang L, Xu K, et al. The Complete Mitochondrial Genome of Mytilisepta virgata (Mollusca: Bivalvia), Novel Gene Rearrangements, and the Phylogenetic Relationships of Mytilidae. Genes [Internet]. 2023; 14(4). doi: 10.3390/genes14040910 37107667 PMC10137486

[59] Xu M, Li J, Guo B, Xu K, Ye Y, Yan X. Insights into the Deep Phylogeny and Novel Gene Rearrangement of Mytiloidea from Complete Mitochondrial Genome. Biochemical Genetics. 2023;61(5):1704–26. doi: 10.1007/s10528-023-10338-4 36745306

[60] Wang Y, Yang Y, Liu H, Kong L, Yu H, Liu S, et al. Phylogeny of Veneridae (Bivalvia) based on mitochondrial genomes. Zoologica Scripta. 2021;50(1):58–70. doi: 10.1111/zsc.12454

[61] Yuan Y, Li Q, Yu H, Kong L. The Complete Mitochondrial Genomes of Six Heterodont Bivalves (Tellinoidea and Solenoidea): Variable Gene Arrangements and Phylogenetic Implications. PLOS ONE. 2012;7(2):e32353. doi: 10.1371/journal.pone.0032353 22384227 PMC3285693

[62] lü J, Dong X, Li J, Ye Y, Xu K. Novel gene re-arrangement in the mitochondrial genome of Pisidia serratifrons (Anomura, Galatheoidea, Porcellanidae) and phylogenetic associations in Anomura. Biodiversity Data Journal. 2023;11:e96231. doi: 10.3897/BDJ.11.e96231 38327357 PMC10848379

[63] Se Sun, Sha Z, Wang Y. The complete mitochondrial genomes of two vent squat lobsters, Munidopsis lauensis and M. verrilli: Novel gene arrangements and phylogenetic implications. Ecology and Evolution. 2019;9(22):12390–407. doi: 10.1002/ece3.5542 31788185 PMC6875667

[64] Yu Y, Kong L, Li Q. Mitogenomic phylogeny of Muricidae (Gastropoda: Neogastropoda). Zoologica Scripta. 2023;52(4):413–25. 10.1111/zsc.12598.

[65] Yang H, Zhang J-e, Luo H, Luo M, Guo J, Deng Z, et al. The complete mitochondrial genome of the mudsnail Cipangopaludina cathayensis (Gastropoda: Viviparidae). Mitochondrial DNA Part A. 2016;27(3):1892–4. doi: 10.3109/19401736.2014.971274 25319293

[66] Tan MH, Gan HM, Lee YP, Bracken-Grissom H, Chan T-Y, Miller AD, et al. Comparative mitogenomics of the Decapoda reveals evolutionary heterogeneity in architecture and composition. Scientific Reports. 2019;9(1):10756. doi: 10.1038/s41598-019-47145-0 31341205 PMC6656734

[67] Kilpert F, Held C, Podsiadlowski L. Multiple rearrangements in mitochondrial genomes of Isopoda and phylogenetic implications. Molecular Phylogenetics and Evolution. 2012;64(1):106–17. doi: 10.1016/j.ympev.2012.03.013 22491068

[68] Bernt M, Bleidorn C, Braband A, Dambach J, Donath A, Fritzsch G, et al. A comprehensive analysis of bilaterian mitochondrial genomes and phylogeny. Molecular Phylogenetics and Evolution. 2013;69(2):352–64. doi: 10.1016/j.ympev.2013.05.002 23684911

[69] Xu W, Jameson D, Tang B, Higgs PG. The Relationship Between the Rate of Molecular Evolution and the Rate of Genome Rearrangement in Animal Mitochondrial Genomes. Journal of Molecular Evolution. 2006;63(3):375–92. doi: 10.1007/s00239-005-0246-5 16838214

[70] Xu T, Qi L, Kong L, Li Q. Mitogenomics reveals phylogenetic relationships of Patellogastropoda (Mollusca, Gastropoda) and dynamic gene rearrangements. Zoologica Scripta. 2022;51(2):147–60. doi: 10.1111/zsc.12524

[71] Zhang Y. Mitochondrial genome rearrangement of Sesarmidae species and its phylogenetic implication [Master]: Zhejiang Ocean University; 2023.

[72] Uribe JE, Kano Y, Templado J, Zardoya R. Mitogenomics of Vetigastropoda: insights into the evolution of pallial symmetry. Zoologica Scripta. 2016;45(2):145–59. 10.1111/zsc.12146.

[73] Osca D, Templado J, Zardoya R. Caenogastropod mitogenomics. Molecular Phylogenetics and Evolution. 2015;93:118–28. doi: 10.1016/j.ympev.2015.07.011 26220836

[74] Osca D, Templado J, Zardoya R. The mitochondrial genome of Ifremeria nautilei and the phylogenetic position of the enigmatic deep-sea Abyssochrysoidea (Mollusca: Gastropoda). Gene. 2014;547(2):257–66. doi: 10.1016/j.gene.2014.06.040 24967939

[75] Choi EH, Hwang UW. The complete mitochondrial genome of an endangered triton snail Charonia lampas (Littorinimorpha: Charoniidae) from South Korea. Mitochondrial DNA Part B. 2021;6(3):956–8. doi: 10.1080/23802359.2021.1889416 33796697 PMC7995882

[76] Zhong S, Huang L, Huang G, Liu Y, Wang W. The first complete mitochondrial genome of MAMMILLA from Mammilla mammata (Littorinimorpha: Naticidae). Mitochondrial DNA Part B. 2020;5(1):96–7. doi: 10.1080/23802359.2019.1698350 33366439 PMC7720982

