# Supplementary material for: Characterization of the complete mitochondrial genome of Desmaulus extinctorium (Littorinimorpha, Calyptraeoidea, Calyptraeidae) and molecular phylogeny of Littorinimorpha
Source: PLoS One. 2024 Mar 28;19(3):e0301389. doi: 10.1371/journal.pone.0301389 (PMC10977763; doi:10.1371/journal.pone.0301389)

PLOS ONE

Characterization of the complete mitochondrial genome of Desmaulus extinctorium (Littorinimorpha, Calyptraeoidea, Calyptraeidae) and molecular phylogeny of Littorinimorpha

--Manuscript Draft--

**Manuscript Number:**	PONE-D-23-34396
**Article Type:**	Research Article
**Full Title:**	Characterization of the complete mitochondrial genome of Desmaulus extinctorium (Littorinimorpha, Calyptraeoidea, Calyptraeidae) and molecular phylogeny of Littorinimorpha
**Short Title:**	Characterization of the complete mitochondrial genome of Desmaulus extinctorium and molecular phylogeny
**Corresponding Author:**	Yingying Ye<br>Zhejiang Ocean University<br>Zhoushan, Zhejiang CHINA
**Keywords:**	Littorinimorpha; Desmaulus extinctorium; gene rearrangement; phylogenetic
**Abstract:**	For the purpose of augmenting the taxonomy and systematics of Calyptraeidae in the evolutionary framework of Littorinimorpha, we hereby furnish a thorough analysis of the mitochondrial genome (mitogenome) sequence of Desmaulus extinctorium. This mitogenome spans 16,605 base pairs and encompasses the entire set of 37 genes, including 13 PCGs, 22 tRNAs and two rRNAs, with an evident AT bias. Notably, tRNASer1 and tRNASer2 lack dihydrouracil (DHU) arms, resulting in an inability to form a secondary structure. Similarly, tRNAAla lacks a TΨC arm, rendering it incapable of forming a secondary structure. In contrast, the remaining tRNAs demonstrate a characteristic secondary structure reminiscent of a cloverleaf. A comparison with ancestral gastropods reveals distinct differences in three gene clusters (or genes), encompassing 15 tRNAs and eight PCGs. Notably, inversions and translocations represent the major types of rearrangements observed in D. extinctorium. Phylogenetic analysis demonstrates robust support for a monophyletic grouping of all Littorinimorpha species, with D. extinctorium forming a distinct Calyptraeoidea clade. In summary, this investigation enhances the entirety of the mitochondrial dataset pertaining to Calyptraeidae, while concurrently providing novel insights into the phylogenetic relationships within Littorinimorpha.
**Order of Authors:**	Yanwen Ma
	Biqi Zheng
	Jiji Li
	Wei Meng
	Kaida Xu
	Yingying Ye
**Additional Information:**	
**Question**	**Response**
**Financial Disclosure**<br><br>Enter a financial disclosure statement that describes the sources of funding for the work included in this submission. Review the submission guidelines for detailed requirements. View published research articles from *PLOS ONE* for specific examples.	Yes

This statement is required for submission and **will appear in the published article** if the submission is accepted. Please make sure it is accurate.<br><br>**Funded studies**<br>Enter a statement with the following details:<br>• Initials of the authors who received each award<br>• Grant numbers awarded to each author<br>• The full name of each funder<br>• URL of each funder website<br>• Did the sponsors or funders play any role in the study design, data collection and analysis, decision to publish, or preparation of the manuscript?<br><br>Did you receive funding for this work?	
Please add funding details.<br>as follow-up to "**Financial Disclosure**<br><br>Enter a financial disclosure statement that describes the sources of funding for the work included in this submission. Review the submission guidelines for detailed requirements. View published research articles from *PLOS ONE* for specific examples.<br><br>This statement is required for submission and **will appear in the published article** if the submission is accepted. Please make sure it is accurate.<br><br>**Funded studies**<br>Enter a statement with the following details:<br>• Initials of the authors who received each award<br>• Grant numbers awarded to each author<br>• The full name of each funder<br>• URL of each funder website<br>• Did the sponsors or funders play any role in the study design, data collection and analysis, decision to publish, or preparation of the manuscript?	This research was financially supported by the National Key R&D Program of China (2019YFD0901204), NSFC Projects of International Cooperation and Exchanges (42020104009).

Powered by Editorial Manager® and ProduXion Manager® from Aries Systems Corporation

Did you receive funding for this work?"	
Please select the country of your main research funder (please select carefully as in some cases this is used in fee calculation).<br>as follow-up to "**Financial Disclosure**<br><br>Enter a financial disclosure statement that describes the sources of funding for the work included in this submission. Review the submission guidelines for detailed requirements. View published research articles from *PLOS ONE* for specific examples.<br><br>This statement is required for submission and **will appear in the published article** if the submission is accepted. Please make sure it is accurate.<br><br>**Funded studies**<br>Enter a statement with the following details:<br>• Initials of the authors who received each award<br>• Grant numbers awarded to each author<br>• The full name of each funder<br>• URL of each funder website<br>• Did the sponsors or funders play any role in the study design, data collection and analysis, decision to publish, or preparation of the manuscript?<br><br>Did you receive funding for this work?"	CHINA - CN
**Competing Interests**<br><br>Use the instructions below to enter a competing interest statement for this submission. On behalf of all authors, disclose any competing interests that could be perceived to bias this work—acknowledging all financial support and any other relevant financial or non-financial competing interests.<br><br>This statement is required for submission	The authors have declared that no competing interests exist.

and **will appear in the published article** if the submission is accepted. Please make sure it is accurate and that any funding sources listed in your Funding Information later in the submission form are also declared in your Financial Disclosure statement.<br><br>View published research articles from *PLOS ONE* for specific examples.<br><br>**NO authors have competing interests**<br><br>Enter: *The authors have declared that no competing interests exist.*<br><br>**Authors with competing interests**<br><br>Enter competing interest details beginning with this statement:<br><br>*I have read the journal's policy and the authors of this manuscript have the following competing interests: [insert competing interests here]*<br><br>* typeset	
**Ethics Statement**<br><br>Enter an ethics statement for this submission. This statement is required if the study involved:<br><br>• Human participants<br>• Human specimens or tissue<br>• Vertebrate animals or cephalopods<br>• Vertebrate embryos or tissues<br>• Field research<br><br>Write "N/A" if the submission does not require an ethics statement.<br><br>General guidance is provided below. Consult the submission guidelines for detailed instructions. **Make sure that all information entered here is included in the Methods section of the manuscript.**	N/A

**Format for specific study types**<br><br>**Human Subject Research (involving human participants and/or tissue)**<br>• Give the name of the institutional review board or ethics committee that approved the study<br>• Include the approval number and/or a statement indicating approval of this research<br>• Indicate the form of consent obtained (written/oral) or the reason that consent was not obtained (e.g. the data were analyzed anonymously)<br><br>**Animal Research (involving vertebrate animals, embryos or tissues)**<br>• Provide the name of the Institutional Animal Care and Use Committee (IACUC) or other relevant ethics board that reviewed the study protocol, and indicate whether they approved this research or granted a formal waiver of ethical approval<br>• Include an approval number if one was obtained<br>• If the study involved *non-human primates*, add *additional details* about animal welfare and steps taken to ameliorate suffering<br>• If anesthesia, euthanasia, or any kind of animal sacrifice is part of the study, include briefly which substances and/or methods were applied<br><br>**Field Research**<br><br>Include the following details if this study involves the collection of plant, animal, or other materials from a natural setting:<br>• Field permit number<br>• Name of the institution or relevant body that granted permission	
**Data Availability**<br><br>Authors are required to make all data underlying the findings described fully available, without restriction, and from the time of publication. PLOS allows rare exceptions to address legal and ethical concerns. See the PLOS Data Policy and FAQ for detailed information.	Yes - all data are fully available without restriction

A Data Availability Statement describing where the data can be found is required at submission. Your answers to this question constitute the Data Availability Statement and **will be published in the article**, if accepted.<br><br>**Important:** Stating 'data available on request from the author' is not sufficient. If your data are only available upon request, select 'No' for the first question and explain your exceptional situation in the text box.<br><br>Do the authors confirm that all data underlying the findings described in their manuscript are fully available without restriction?	
**Describe where the data may be found in full sentences. If you are copying our sample text, replace any instances of XXX with the appropriate details.**<br><br>• If the data are **held or will be held in a public repository**, include URLs, accession numbers or DOIs. If this information will only be available after acceptance, indicate this by ticking the box below. For example: *All XXX files are available from the XXX database (accession number(s) XXX, XXX.*).<br>• If the data are all contained **within the manuscript and/or Supporting Information files**, enter the following: *All relevant data are within the manuscript and its Supporting Information files.*<br>• If neither of these applies but you are able to provide **details of access elsewhere**, with or without limitations, please do so. For example:<br><br>*Data cannot be shared publicly because of [XXX]. Data are available from the XXX Institutional Data Access / Ethics Committee (contact via XXX) for researchers who meet the criteria for access to confidential data.*<br><br>*The data underlying the results presented in the study are available from (include the name of the third party*	The datasets analysed during the current study are available in the National Center for Biotechnology Information, and the GenBank accession numbers of the mtgenome of D. extinctorium is OQ511529

and contact information or URL).<br>• This text is appropriate if the data are owned by a third party and authors do not have permission to share the data.<br><br>* typeset	
Additional data availability information:	Tick here if your circumstances are not covered by the questions above and you need the journal’s help to make your data available.

Powered by Editorial Manager® and ProduXion Manager® from Aries Systems Corporation

Click here to access/download;Manuscript;Manuscript (PLos One).docx

Characterization of the complete mitochondrial genome of *Desmaulus extinctorium* (Littorinimorpha, Calyptraeoidea, Calyptraeidae) and molecular phylogeny of Littorinimorpha

Yanwen Ma [1], Biqi Zheng [3], Jiji Li [1], Wei Meng [2], Kaida Xu [2,*] and Yingying Ye [1,*]

1 National Engineering Research Center for Marine Aquaculture, Zhejiang Ocean University, Zhoushan 316022, China, 2 Key Laboratory of Sustainable Utilization of Technology Research for Fisheries Resources of ZhejiangProvince, Scientific Observing and Experimental Station of Fishery Resources for Key Fishing Grounds,Ministry of Agriculture and Rural Affairs of China, Zhejiang Marine Fisheries Research Institute, Zhoushan 316021, China, 3 Ningde Marine Center, Department of Natural Resources, Ningde 200136, China,

* h01011@zjou.edu.cn (KX); yeyy@zjou.edu.cn (YY)

Abstract

For the purpose of augmenting the taxonomy and systematics of Calyptraeidae in the evolutionary framework of Littorinimorpha, we hereby furnish a thorough analysis of the mitochondrial genome (mitogenome) sequence of *Desmaulus extinctorium*. This mitogenome spans 16,605 base pairs and encompasses the entire set of 37 genes, including 13 PCGs, 22 tRNAs and two rRNAs, with an evident AT bias. Notably, $tRNA^{Ser1}$ and $tRNA^{Ser2}$ lack dihydrouracil (DHU) arms, resulting in an inability to form a secondary structure. Similarly, $tRNA^{Ala}$ lacks a TΨC arm, rendering it incapable of forming a secondary structure. In contrast, the remaining tRNAs demonstrate a characteristic secondary structure reminiscent of a cloverleaf. A comparison with ancestral gastropods reveals distinct differences in three gene clusters (or genes), encompassing 15 tRNAs and eight PCGs. Notably, inversions and translocations represent the major types of rearrangements observed in *D. extinctorium*. Phylogenetic analysis demonstrates robust support for a monophyletic grouping of all Littorinimorpha species, with *D. extinctorium* forming a distinct Calyptraeoidea clade. In summary, this investigation enhances the entirety of the mitochondrial dataset pertaining to Calyptraeidae, while concurrently providing novel insights into the phylogenetic relationships within Littorinimorpha.

Keywords: Littorinimorpha; *Desmaulus extinctorium*; gene rearrangement; phylogenetic

Introduction

Mitochondria are double - membrane - coated organelles found in most eukaryotes. Although most of a cell's DNA is contained in the nucleus, mitochondria have their own genome, known as the mitogenome. Attributable to its profoundly conserved characteristics, absence of extensive recombination, maternal inheritance, and elevated mutation rate [1-3], the mitogenome has found extensive utility in the realms of comparative and evolutionary genomics [4], species identification, population genetics [5], molecular evolution, and phylogenetic relationships [6,7]. In particular, phylogeny based on complete mitochondrial genomes have demonstrated improved resolution compared to phylogenetic trees inferred from partial gene fragments such as *COI* and *16S rRNA* [8]. In recent years, mitochondrial genome sequencing and amplification techniques have rapidly developed, and mitochondrial genomes have been extensively utilized to reconstruct phylogenetic trees of different gastropods. For instance, Yang et al [9] sequenced the complete mitochondrial genomes of nine Nassariidae species and compared them with eight previously reported Nassariidae genomes, identifying the phylogenetic placement of these nine species within the gastropod clade. Genetic distance analysis and phylogenetic analysis both supported ~~that~~ the distant relationship of *Nassarius jacksonianus* and *Nassarius acuticostus* to other *Nassarius* members. Furthermore, Yang et al [10] sequenced the complete mitochondrial genomes of two Nassariids (Neogastropoda: Nassariidae: *Nassarius*), *Nassarius glans* and *Nassarius siquijorensis*, identifying the phylogenetic positions of these two species within *Nassarius*. In addition, Lee et al [11] ~~investigated~~ the complete mitochondrial genome of *Semisulcospira gottschei* (Gastropoda: Caenogastropoda) ~~to identify~~ its phylogenetic relationship within ~~gastropod species~~. The

study revealed that *Semisulcospira gottschei* ~~is the closest relative to~~ *Semisulcospira coreana*, and it was classified within the family Cerithioidea.

Desmaulus extinctorium is a marine snail that inhabits geological mud at depths of around nine meters ~~deep~~. It belongs to the class Gastropoda, subclass Caenogastropoda, order Littorinimorpha, superfamily Calyptraeoidea, family Calyptraeidae, genus *Desmaulus*. *D. extinctorium* is primarily found in the southern China and Hongkong [12]. ~~While~~ previous research on this family has predominantly focused on morphology and growth [13-15]. Calyptraeid gastropods are known for their taxonomic challenges stemming from their simple, phenotypically variable shells [16]. As such, only a few studies have explored the phylogenetic analysis of this family. For instance, Regina conducted sequencing on a segment of the mitochondrial genome from the calyptraeoidean species *Calyptraea chinensis*, which belongs to the Littorinimorpha. Phylogenetic investigations have revealed that the Littorinimorpha does not form a monophyletic cluster [17]. Meanwhile, Rachel examined how development modes influence the phylogeography and population dynamics of North Atlantic *Crepidula* (Gastropoda: Calyptraeidae). She created haplotype trees for each clade using 640 bp *COI* sequences. Examination of both the tree topology and AMOVA revealed that species undergoing direct development (hatching as benthic juveniles) displayed a more conspicuous population structure in comparison to those species undergoing planktonic development [18]. Prior to our study, a complete mitochondrial genome of Calyptraeidae had not been uploaded to GenBank.

Littorinimorpha is a substantial family within Caenogastropoda (Class Gastropoda), encompassing 16 superfamilies according to the WoRMS database. Among marine snails, Caenogastropoda stands as the dominant group in terms of species numbers, diversity of habitats, ecological importance and

behaviors. The current classification of Littorinimorpha was mainly established by Bouchet [19]. While Colgan et al. [20] conducted an exhaustive phylogenetic investigation of Caenogastropoda, the interrelationships among families and superfamilies within the Caenogastropoda clade remain predominantly unresolved. The monophyly of both Littorinimorpha and Neogastropoda has been a topic of ongoing debate [21]. Regina et al. [17] conducted the sequencing of complete mitochondrial genomes for seven previously unanalyzed gastropod species. Subsequent phylogenetic analysis led to the rejection of the monophyletic status of Neogastropoda, attributed to the incorporation of Littorinimorpha lineages within this cluster. Additiongally, Zhao et al. [22] sequenced the complete mitochondrial genomes of intermediate host snails for Schistosoma and performed a phylogenetic analysis, revealing that neither Neogastopoda nor Littorinimorpha were monophyletic groups. Consequently, further research is necessary to refine the phylogenetic relationship within Caenogastropoda. To shed light on the phylogenetic relationships of Littorinimorpha, Wang et al. [23] sequenced the complete mitochondrial genomes of *Ficus* ~~*variegate*~~ (Littorinimorpha: Ficoidea) and performed phylogenetic analysis. They found that *F. variegate* clustered with the the Tonnoidea, attributable to the historical placement of Ficoidea within the superfamily Tonnoidea as Ficidae. Riedel [24] later reclassified figs from Tonnoidea in 1994 and established superfamily Ficoidea. And then, Jiang et al. [25] reconstructed the phylogenetic tree of Littorinimorpha by sequencing the complete mitochondrial genome of two species in the Stromboidea. The findings provided evidence for the existence of three significant clades within Littorinimorpha: 1) Stromboidea, Tonnoidea, Littorinoidea, and Naticoidea, 2) Rissooidea alongside Truncatelloidea, and 3) Vermetoidea

In this investigation, we have accomplished the comprehensive sequencing of the mitogenome for *D. extinctorium*. Furthermore, an elucidation of the gene structure within the mitogenome of *D. extinctorium* has been presented, coupled with a phylogenetic scrutiny encompassing 51 species from the Littorinimorpha taxon. This analysis is predicated upon the nucleotide sequences of 13 PCGs. As an outcome of this study, there has been an augmentation of the mitochondrial genome repertoire for Littorinimorpha, along with the provision of data requisite for subsequent phylogenetic assessments within the Littorinimorpha clade.

Materials and Methods

Sampling and DNA extraction

We obtained a specimen of *D. extinctorium* from Ningde, Fujian Province, China (27°04′812N, 120°24′158″E). The initial morphological classification of these samples involved expert consultation with taxonomists at Zhejiang Ocean University's Marine Biological Museum. After collection, the specimen was rapidly submerged in absolute ethanol and stored at -20°C. To confirm its classification, we relied on morphological traits, and we preserved fresh tissues in absolute ethanol before DNA extraction. We used the salt-extraction technique [26] to isolate complete genomic DNA, which was then stored at -20°C.

Genome sequencing, assembly and annotation

The mitogenomes of *D. extinctorium* were sequenced by Origin gene Co. Ltd., situated in Shanghai, China, employing the Illumina HiSeq X Ten sequencing platform. HiSeq X Ten libraries were prepared, incorporating an insert size ranging from 300 to 500 base pairs, sourced from genomic DNA samples. Each library yielded approximately 10 gigabases of raw data. Preprocessing procedures encompassed

the elimination of low-quality reads, adapters, sequences containing high proportions of ambiguous bases ("N" bases), and those with a length below 25 base pairs. For assembly, the NOVOPlasty software [27] (accessible at https://github.com/ndierckx/NOVOPlasty) was utilized. Annotation and manual refinement of the assembly were performed with reference to established mitogenome datasets. De novo assembled mitogenomes were generated using MITOS tools [28] (accessed through the MITOS Web Server at uni-leipzig.de). Validation of sequence accuracy was achieved through alignment against mitochondrial genes of other Calyptraeoidea species, complemented by confirmation via the COI barcode sequence and NCBI BLAST searches [29].

Reads were reconstructed using a de novo assembly program, and subsequent annotation of complete mitogenomes was conducted using Sequin version 16.0. The mitogenome map of *D. extinctorium* was visualized utilizing the online tool Poksee (accessible at https://proksee.ca) [30]. Secondary structures of tRNA genes were forecasted and illustrated through the MITOS Web Server. To gain insights into coding sequence characteristics, relative synonymous codon usage (RSCU) values and substitution saturation for the 13 protein-coding genes (PCGs) were computed utilizing DAMBE 5. Subsequent analysis of these values was executed using MEGA 7 [31]. Additionally, base compositional disparities and strand asymmetry among samples were assessed by evaluating GC-skews and AT-skews. These parameters were calculated using the following formulas: AT-skew = [A−T]/[A+T] and GC skew = [G−C]/[G+C]. Substitution saturation for the 13 PCGs was quantified using DAMBE 5 [32].

Gene Order Analysis

In addition to the mitogenomes sequenced in this study, we obtained an additional 51 complete mitogenomes of Littorinimorpha from GenBank (Table 1) for comparative analyses. The gene

arrangements of all 51 mitogenomes were compared with the ancestral Gastropoda, with the aim of identifying potential novel gene orders that have not been reported in previous studies. To ensure that observed gene order differences were not caused by mis-annotations, any mitogenomes in Littorinimorpha that deviated from the ancestral pattern underwent re-annotation using MITOS [28].

Table 1. List of species of Littorinimorpha analysed in this study and their GenBank accession numbers

Superfamily	Family	Species	Accession no.	Size(bp)
Stromboidea	Strombidae	*Aliger gigas*	MZ157283	15460
		Conomurex luhuanus	NC_035726	15799
		Harpago chiragra	MN885884	16404
		Laevistrombus canarium	MT937083	15626
		Lambis lambis	MH115428	15481
		Strombus pugilis	MW244819	15809
		Tridentarius dentatus	MW244820	15500
	Aporrhaidae	*Aporrhais serresiana*	MW244817	15455
	Struthiolariidae	*Struthiolaria papulosa*	MW244818	15475
	Seraphsidae	*Terebellum terebellum*	MW244821	15478
	Rostellariidae	*Tibia fusus*	NC_065371	16083
		Varicospira cancellata	MW244822	15864
	Xenophoridae	*Xenophora japonica*	MW244823	15684
Truncatelloidea	Amnicolidae	*Baicalia turriformis*	NC_035869	15127
		Godlewskia godlewskii	NC_035870	15224
		Maackia herderiana	NC_035871	15154
	Pomatiopsidae	*Oncomelania hupensis*	NC_013073	15182
		Tricula hortensis	NC_013833	15179
	Tateidae	*Potamopyrgus antipodarum*	NC_070577	16846
		Potamopyrgus estuarinus	NC_070576	16701
Tonnoidea	Bursidae	*Bufonaria rana*	MT408027	15510
	Charoniidae	*Charonia lampas*	KU237290	15330
		Charonia tritonis	MT043269	15346
	Cassidae	*Galeodea echinophora*	NC_028003	15388
	Cymatiidae	*Monoplex parthenopeus*	NC_013247	15270
Naticoidea	Naticidae	*Cryptonatica andoi*	NC_046598	15302
		Cryptonatica janthostoma	NC_046704	15235
		Euspira gilva	NC_046593	15315
		Euspira pila	NC_046703	15244

		Glossaulax reiniana	NC_041162	15254
		Mammilla mammata	NC_046597	15319
		Mammilla kurodai	NC_046596	15309
		Naticarius hebraeus	NC_028002	15384
		Neverita didyma	NC_046594	15629
		Notocochlis gualteriana	NC_046705	15176
		Paratectonatica tigrina	NC_050661	15201
		Polinices sagamiensis	NC_046595	15383
		Tanea lineata	NC_050662	15156
Cypraeoidea	Cypraeidae	*Cypraea tigris*	MK783263	16177
		Erronea errones	NC_066082	15422
Vermetoidea	Vermetidae	*Dendropoma gregarium*	NC_014580	15641
		Eualetes tulipa	NC_014585	15078
		Thylacodes squamigerus	NC_014588	15544
Ficoidea	Ficidae	*Ficus variegata*	NC_056153	15736
Littorinoidea	Littorinidae	*Littoraria ardouiniana*	NC_066085	16261
		Littoraria intermedia	NC_064397	16194
		Littoraria melanostoma	NC_064398	16149
		Littoraria sinensis	MN496138	16420
		Littorina brevicula	NC_050987	16356
		Littorina saxatilis	NC_030595	16887
		Melarhaphe neritoides	MH119311	15676
Calyptraeoidea	Calyptraeidae	*Desmaulus extinctorium*	OQ511529	16572
Outgroup		*Donax variegatus*	NC_035986	17195
		Donax vittatus	NC_035987	17070

Phylogenetic analysis

Exploring the evolutionary relationships within the Littorinimorpha clade involved an analysis of 13 PCGs. These genes were sourced from a comprehensive dataset that included 51 complete mitogenome sequences. The mitogenome sequences were retrieved from the GenBank database (https://www.ncbi.nlm.nih.gov/genbank/). To provide additional context, two species from the Donacidae family were also included as representatives of the outgroup. The assessment of phylogenetic

relationships utilized both Maximum Likelihood (ML) and Bayesian Inference (BI) methodologies [33-35].

The ML analysis, carried out with IQ-TREE 1.6.2, involved 1000 ultrafast likelihood bootstrap replicates. The choice of optimal models was guided by the Bayesian Information Criterion (BIC), leading to the adoption of the GTR + F + R6 model for each partition. We conducted Bayesian Inference (BI) analyses using the MrBayes 3.2 software, and model selection was facilitated by MrMTgui [36], a tool that connects PAUP, ModelTest, and MrModelTest across different platforms. For model selection, we chose the best-fit model (GTR + I + G) based on AIC results obtained from MrModelTest 2.3 [37]. Bayesian analyses were then performed in MrBayes, utilizing parameter values from either MrModelTest or ModelTest (nst = 6, rates = invgamma) [38]. The Bayesian analyses utilized Markov Chain Monte Carlo (MCMC) sampling, involving two independent runs of three hot chains and one cold chain. These chains ran simultaneously for 2,000,000 generations, with sampling intervals set at 1000 steps and a relative burn-in rate of 25%. We assessed the convergence of independent runs by examining the mean standard deviation of split frequencies (< 0.01). Finally, the resulting phylogenetic trees were visualized and edited using Figure Tree v.1.4.3 software [39].

Results discussion

Genome structure and composition

The complete mitogenome sequence of *D. extinctorium* constitutes a prototypical closed-circular molecule spanning 16,605 bp in length (GenBank accession number OQ511529). This genome encompasses a total of 37 genes, comprising 13 protein-coding genes (PCGs), 22 transfer RNAs (tRNAs), two ribosomal RNAs (*16S rRNA* and *12S rRNA*), and a concise non-coding region. This structural

arrangement aligns consistently with the composition observed in the majority of previously investigated mollusks [40-42]. All these genes have been discerned and are depicted in Figure 1 and Table 2. Among the 37 genes, the majority are localized on the heavy (H-) strand, except for eight tRNAs (*tRNA-Phe*, *His*, *Pro*, *Leu*, *Val*, *Gln*, *Cys*, and *Tyr*). (Fig 1. Maps of the mitochondrial genomes of *D. extinctorium*.)

Table 2. Mitochondrial genome organization of *D. extinctorium*.

Gene	Direction	Position	Length/bp		Start/Stop codon	Intergenic Nucleotide(bp)	Anticodon
COX1	H	1	1551	1551	ATT/TAA	22	
COX2	H	1574	2266	693	ATG/TAA	46	
$tRNA^{Asp}$	H	2313	2382	70		82	GTC
ATP8	H	2465	2623	159	ATG/TAA	57	
ATP6	H	2681	3376	696	ATG/TAA	26	
$tRNA^{Met}$	L	3403	3468	66		8	CAT
$tRNA^{Tyr}$	L	3477	3542	66		4	GTA
$tRNA^{Cys}$	L	3547	3612	66		0	GCA
$tRNA^{Trp}$	L	3613	3679	67		-2	TCA
$tRNA^{Gln}$	L	3678	3743	66		4	TTG
$tRNA^{Gly}$	L	3748	3813	66		-1	TCC
$tRNA^{Glu}$	L	3813	3882	70		80	TTC
12S rRNA	H	3963	4858	896		-1	
$tRNA^{Val}$	H	4858	4925	68		-10	TAC
16S rRNA	H	4916	6279	1364		13	
$tRNA^{Leu1}$	H	6293	6364	72		4	TAG
$tRNA^{Leu2}$	H	6369	6438	70		0	TAA
NAD1	H	6439	7383	945	ATG/TAA	12	
$tRNA^{Pro}$	H	7396	7463	68		6	TGG
NAD6	H	7470	7973	504	ATG/TAA	16	
Cytb	H	7990	9129	1140	ATG/TAA	17	
$tRNA^{Ser2}$	H	9147	9212	66		0	TGA
NAD4l	H	9213	9515	303	ATG/TAG	80	
NAD4	H	9596	10900	1305	ATG/TAA	10	
$tRNA^{His}$	H	10911	10976	66		0	GTG
NAD5	H	10977	12848	1872	ATG/TAG	10	
$tRNA^{Phe}$	H	12859	12924	66		12	GAA
$tRNA^{Thr}$	L	13573	13640	68		104	TGT

COX3	H	13745	14524	780	ATG/TAA	29	
$tRNA^{Lys}$	H	14554	14628	75		13	TTT
$tRNA^{Ala}$	H	14684	14734	51		17	TGC
$tRNA^{Arg}$	H	14752	14821	70		21	TCG
$tRNA^{Asn}$	H	14843	14912	70		23	GTT
$tRNA^{Ile}$	H	14936	15005	70		0	GAT
NAD3	H	15010	15366	357	ATG/TAG	1	
$tRNA^{Ser1}$	H	15416	15483	68		2	GCT
NAD2	H	15484	16572	1089	ATG/TAA	36	

The longest gene, *ND5*, stretches across 1872 base pairs, whereas the shortest is $tRNA^{Ala}$, comprising a mere 51 base pairs. The *D. extinctorium* mitogenome comprises four regions displaying overlap. Of these, one involves a 10 bp overlap with $tRNA^{Val}$, and the remaining three exhibit overlaps shorter than 10 bp with $tRNA^{Trp}$ (2 bp), $tRNA^{Gly}$ (1 bp), and *16S rRNA* (1 bp). Additionally, the *D. extinctorium* mitogenome accommodates 1393 bp of intergenic spacers distributed across 28 regions, ranging in size from 3 to 648 bp (Table 2).

Table 3. Nucleotide contents of the coding and non-coding regions of the mitochondrial genome of *D. extinctorium*, indicating AT-, GC-skew ratios.

Region	Size(bp)	A (%)	T (%)	G (%)	C (%)	A+T (%)	AT-skew	GC-skew
Mitogenome	16608	27.73	42.47	18.08	11.72	70.20	-0.210	0.213
COX1	1551	23.92	43.13	20.05	12.89	67.05	-0.287	0.217
COX2	693	27.13	38.96	20.49	13.42	66.09	-0.179	0.209
ATP8	159	27.67	44.65	15.72	11.95	72.32	-0.235	0.136
ATP6	696	23.13	46.70	16.67	13.51	69.83	-0.337	0.105
COX3	780	21.67	42.31	22.31	13.72	63.98	-0.323	0.238
NAD3	357	19.89	47.90	20.73	11.48	67.79	-0.413	0.287
NAD1	945	24.02	45.29	18.20	12.49	69.31	-0.307	0.186
NAD5	1872	26.82	42.63	16.61	13.94	69.45	-0.228	0.087
NAD4	1305	25.21	46.13	16.86	11.80	71.34	-0.293	0.176
NAD4l	303	26.73	43.89	19.80	9.57	70.62	-0.243	0.348
NAD6	504	26.39	46.43	18.65	8.53	72.82	-0.275	0.372
Cytb	1140	24.39	44.39	17.81	13.42	68.78	-0.291	0.140
NAD2	1089	25.34	46.37	18.64	9.64	71.71	-0.293	0.318

tRNAs	1485	34.61	35.62	17.24	12.53	70.23	-0.014	0.158
rRNAs	2260	34.91	38.14	17.08	9.87	73.05	-0.044	0.268
PCGs	11394	24.84	44.25	18.47	12.44	69.09	-0.281	0.195

Regarding nucleotide composition, the *D. extinctorium* mitogenome is comprised of A (27.73%), T (42.47%), G (18.08%), and C (11.71%), demonstrating a conspicuous AT bias. These findings parallel not only those observed in numerous mollusks [43,44] but also in certain crustaceans like crabs and lobsters [45,46]. The cumulative A + T (%) content of the mitogenomes stands at 70.20%. Calculated for the selected complete mitogenomes, the AT-skew of the *D. extinctorium* mitogenome is negative (−0.210), while the GC-skew is positive (0.214), implying a higher abundance of Ts and Cs than As and Gs. These outcomes align with those identified in specific Neritidae species [40].

Transfer RNAs, ribosomal RNAs

Similar to the prevailing pattern in many invertebrate species [47,48], the mitogenome of *D. extinctorium* harbors a total of 22 tRNA genes. Among these, fourteen are encoded by the heavy strand (H-), while the remaining ones are encoded by the light strand (L-). Across the entire mitogenome, the size of tRNA molecules spans from 51 to 75 bp, collectively encompassing a length of 1485 bp, characterized by a pronounced AT bias (70.23%). The AT-skew and GC-skew values are recorded as –0.014 and 0.158, respectively, signifying a subtle inclination towards adenine usage and a conspicuous predilection for guanine usage (Table 3). The $tRNA^{Ser1}$ and $tRNA^{Ser2}$, due to the absence of dihydrouracil (DHU) arms, along with $tRNA^{Ala}$, due to the lack of a TΨC arm, are unable to adopt a secondary structure. Conversely, other tRNAs possess the capacity to fold into a conventional clover-leaf secondary structure. Notably, the structural variation observed in $tRNA^{Ser1}$ corresponds with the $tRNA^{Ser1}$ configuration documented in other invertebrate mitogenomes [49]. Moreover, G-C mismatches are evident in all

tRNAs except *tRNA*^{Leu2}, *tRNA*^{Met}, *tRNA*^{Trp}, and *tRNA*^{Tyr}. (Fig 2. Secondary structure of the tRNA genes in the mitogenome of *D. extinctorium*. The tRNAs are labeled with the abbreviations of their corresponding amino acids. Blue dots indicate normal conditions and yellow dots indicate base mismatches.).

The sizes of the *12S rRNA* and *16S rRNA* components are 896 bp and 1364 bp, respectively, typically demarcated by *tRNA*^{Val} (Table 2). These dimensions align comparably with those observed in other invertebrate species. The A-T content of the rRNAs is determined to be 73.05%. AT-skew and GC-skew values are recorded as – 0.044 and 0.268, respectively, indicating a modest tendency towards adenine utilization and a marked preference for guanine utilization (Table 3). The control region (CR), positioned between *tRNA*^{Thr} and *tRNA*^{Phe}, spans a length of 648 bp.

Table 4. Relative synonymous codon usage (RSCU) in the mitogenomes of *D. extinctorium*.

Codon	Count	RSCU	Codon	Count	Codon	Codon	Count	RSCU	Codon	Count	Co-don
GCU(A)	84.0	2.11	CCU(P)	64.0	2.08	AGA(S)	85.0	1.17	CAU(H)	57.0	1.54
GCC(A)	19.0	0.48	CCC(P)	19.0	0.62	AGG(S)	83.0	1.14	CAC(H)	17.0	0.46
GCA(A)	41.0	1.03	CCA(P)	27.0	0.88	AUU(I)	293.0	1.70	ACU(T)	97.0	2.38
GCG(A)	15.0	0.38	CCG(P)	13.0	0.42	AUC(I)	52.0	0.30	ACC(T)	20.0	0.49
UGU(C)	123.0	1.43	CAA(Q)	53.0	1.15	AAA(K)	179.0	1.39	ACA(T)	34.0	0.83
UGC(C)	49.0	0.57	CAG(Q)	39.0	0.85	AAG(K)	79.0	0.61	ACG(T)	12.0	0.29
GAU(D)	112.0	1.75	CGU(R)	35.0	1.77	UUA(L)	336.0	2.66	GUU(V)	182.0	2.25
GAC(D)	16.0	0.25	CGC(R)	5.0	0.25	UUG(L)	160.0	1.27	GUC(V)	33.0	0.41
GAA(E)	95.0	1.43	CGA(R)	18.0	0.91	CUU(L)	126.0	1.00	GUA(V)	66.0	0.82
GAG(E)	38.0	0.57	CGG(R)	21.0	1.06	CUC(L)	26.0	0.21	GUG(V)	42.0	0.52
UUU(F)	512.0	1.65	UCU(S)	113.0	1.55	CUA(L)	67.0	0.53	UGA(W)	115.0	1.11
UUC(F)	108.0	0.35	UCC(S)	44.0	0.60	CUG(L)	43.0	0.34	UGG(W)	92.0	0.89
GGU(G)	102.0	1.47	UCA(S)	83.0	1.14	AUA(M)	135.0	1.19	UAU(Y)	240.0	1.56
GGC(G)	41.0	0.59	UCG(S)	31.0	0.43	AUG(M)	91.0	0.81	UAC(Y)	68.0	0.44
GGA(G)	65.0	0.94	AGU(S)	100.0	1.37	AAU(N)	189.0	1.65	UAA(*)	174.0	1.25
GGG(G)	70.0	1.01	AGC(S)	43.0	0.59	AAC(N)	40.0	0.35	UAG(*)	105.0	0.75

PCGs and codon usage

The result presents the initiation and termination codons for all Protein-Coding Genes (PCGs) within *D. extinctorium* in table 3. The mitochondrial genome of D. extinctorium encompasses a total of 13 PCGs, comprising a cytochrome b (*Cytb*), two ATPases (*ATP6* and *ATP8*), three cytochrome oxidases (*COI–III*), and seven NADH dehydrogenases (*ND1–6* and *ND4L*). This configuration aligns with the established structural pattern observed in the Muricidae family [47]. The collective length of these 13 PCGs amounts to 11,484 bp. Within this set, the individual PCGs exhibit a range of lengths spanning from 159 to 1,872 bp. Notably, the average A+T content stands at 69.13%, with variations across the spectrum from 63.98% (*COIII*) to 72.82% (*ND6*) (Table 2). The AT-skew and GC-skew values are calculated as -0.281 and 0.195, respectively (Table 4). It is noteworthy that all PCGs commence with the initiation codon ATG, except for *ND4*, which employs ATT as its start codon. Furthermore, the majority of PCGs terminate with TAA, whereas *ND4L*, *ND5*, and *ND5* employ TAG as their respective stop codons (Table 4). Examining the amino acid utilization in *D. extinctorium*, $tRNA^{Phe}$ emerges as the most frequently employed, while $tRNA^{His}$ is the least prevalent (Figure 2). Relative synonymous codon usage (RSCU) values for the 13 PCGs in *D. extinctorium* are presented in Table 4 and Figure 3. Among these, UUA (Leu) ranks as the most frequently encountered codon, whereas CUC (Leu) stands as the least common codon. (Fig 3. Codon usage patterns in the mitogenome of *D. extinctorium*. CDspT, codons per thousand codons. Codon families are provided on the x-axis (A) and the relative synonymous codon usage (RSCU) (B)).

Gene re-arrangement

Rearrangements in mitochondrial gene order present an autonomous dataset for resolving evolutionary relationships. Shared patterns of mitogenome gene order rearrangements among distinct taxonomic groups are likely indicative of common ancestry rather than products of convergent evolution [6,50]. In comparison to the ancestral gastropod gene arrangement, significant rearrangements are evident in the mitogenome of *D. extinctorium*. As illustrated in Figure 4, a minimum of three gene clusters (or genes) differ notably from the conventional arrangement, encompassing 15 tRNA genes (*M*,

Y, C, W, Q, G, E, V, L, P, S, H, F, and *T*), as well as eight protein-coding genes (*16S rRNA, 12S rRNA, NAD1, NAD6, Cytb, NAD4L, NAD4,* and *NAD5*). The rearrangement of these three gene clusters (or genes) is detailed as follows (Figure 4): (1) The *M-Y-C-W-Q-G-E* cluster has relocated downstream of *ATP6*; (2) The *T* cluster has shifted downstream of *F*; (3) The *F-ND5-H-ND4-ND4L-S-Cytb-ND6-P-ND1-L-16S-V-12S* underwent inversion and translocation. (Fig 4. Comparison of mitochondrial gene rearrangements of the *D. extinctorium*. The green squares represent PCGs, the yellow squares represent tRNAs, and the orange squares represent rRNAs. The position at the top indicates that it is encoded in the H chain, and the position at the bottom indicates that it is encoded in the L chain.)

Furthermore, mitochondrial gene rearrangements have frequently been linked to heightened rates of evolution [51]. Prior investigations have identified a notable positive correlation in mitochondrial genomes between rates of gene order rearrangement and accelerated evolutionary rates [52]. Intriguingly, when compared to the extensive gene rearrangements observed in Lottiidae, Littorinimorpha exhibits minimal differences in genetic order, with the exception of Vermetoidea [53]. We postulate that this circumstance could be attributed to the relatively modest variations in genome size among Littorinimorpha species, ranging from 15,127 bp to 17,195 bp (Tab 1), while the mitochondrial genome size within Lottiidae spans from 16,319 bp to 26,835 bp. Further investigations are warranted to scrutinize this association within a broader spectrum of Gastropoda groups.

In the context of gene rearrangement patterns, three primary categories are recognized [54]: (1) shuffling, where genes migrate from their original locations to adjacent positions on the same strand, typically without traversing protein-coding genes; (2) translocation, in which genes traverse several genes, often including protein-coding genes, relocating from their original positions to new sites; (3)

inversion, involving the switch of genes from one strand to the other. Based on the characteristics of mitochondrial sequences, our analysis suggests that inversion and translocation are the predominant types of rearrangements observed in D. extinctorium.

Furthermore, we conducted a comparative examination of the gene order in *D. extinctorium* against other superfamilies within Littorinimorpha. With the exception of Vermetoidea, the gene order across other superfamilies remains largely consistent. Notably, in Vermetoidea, significant deviations in ~~genetic~~ order primarily pertain to tRNAs. In addition, the *M-Y-C-W-Q-G-E* cluster within the mitochondrial genome of Vermetoidea has undergone inversion, a phenomenon observed in other gastropod mitochondrial genomes [55], resulting in disruption and rearrangement. Intriguingly, a remarkably similar set of genes undergoes rearrangement in the common ancestor of Caenogastropoda, although the integrity of the *M-Y-C-W-Q-G-E* cluster is maintained [17, 21, 56]. These findings align with the conclusions drawn from gene order-based phylogenetic analysis, underscoring the utility of comparing mitochondrial gene rearrangements as a valuable tool in phylogenetic studies.

Phylogenetic relationships

In this current study, we conducted an analysis of phylogenetic relationships using the sequences of 13 protein-coding genes (PCGs). The primary objective was to gain insights into the interrelationships within the Littorinimorpha clade, focusing on *D. extinctorium.* Additionally, we included 51 other well-known Littorinimorpha species in our analysis, with *Donax variegatus* and *Donax vittatus* serving as outgroups. Both the Maximum Likelihood (ML) tree and the Bayesian Inference (BI) tree revealed consistent topological structures, although they exhibited varying degrees of support values. Notably, BI generally yielded higher support values, with most nodes having a support value of 1. In contrast, the

support values in ML, except for three nodes in the Stromboidea superfamily, were below 70, and the majority of other branches had support values above 90. Consequently, we present and display only one topology (ML) with both support values. (Fig 5. The phylogenetic tree was inferred from the nucleotide sequences of 13 mitogenome PCGs using BI and ML methods. Numbers on branches indicate posterior probability (BI) and bootstrap support (ML)).

Among the 19 families encompassed within our phylogenetic tree, each individual family constitutes a monophyletic clade, bolstered by elevated nodal support values. Phylogenetic analysis showed that nine superfamilies within the Littorinimorpha show the following relationship: ((((((Naticoidea + Littorinoidea) + Truncatelloidea) + Tonnoidea + Ficoidea + Cypraeoidea) +Stromboidea) + Calyptraeoidea) + Vermetoidea), and all nine of them are monophyletic groups, some previous studies have shown that this is plausible [57, 58], and Naticoidea and Littorinoidea are the closest sisters to each other. Additionally, phylogenetic tree showed that (Tonnoidea + Ficoidea + Cypraeoidea) formed a clade which showing were sister groupsin this tree, while *D. extinctoriu*m alone forms a Calyptraeoidea clade, and (Calyptraeoidea + (Stromboidea + (Tonnoidea + Ficoidea + Cypraeoidea) + Truncatelloidea + (Naticoidea + Littorinoidea))) formed a clade. Vermetoidea is placed at the basal position of the monophyletic Littorinimorpha, this is consistent with previous research [21], and this can also be related to the results of gene re-arrangements, only the Vermetoidea has a significantly different genetic sequence from the rest of the species, so Vermetoidea is at the bottom of the phylogenetic tree. Stromboidea is the superfamily containing the largest number of families, it is a highly diverse group. Stromboidea is currently understood to comprise six recent families: Aporrhaidae, Rostellariidae, Seraphsidae, Strombidae, Struthiolariidae and Xenophoridae. At the family level, species

of each family formed a distinct clade. Phylogenetic relationships in the superfamily can be seen from the branches, Strombidae, Seraphsidae and Rostellariidae have a close kinship, Aporrhaidae, Struthiolariidae and Xenophoridae have a close kinship, the result consistent with the findings of Alison R. Irwin [59]. Naticidae is the family that contains the most species and occupied the most branches in the evolutionary tree, and Littorinidae is sister group of Naticidae which is also a large family.

Conclusion

In this investigation, we conducted the sequencing of the mitogenome of *D. extinctorium* employing next-generation sequencing techniques, thus yielding novel mitochondrial data pertinent to Calyptraeidae. An exhaustive examination of the mitogenome of *D. extinctorium* revealed its substantial resemblance to other representatives of the Littorinimorpha order, characterized by several notable features, including AT-skew and a codon usage bias, among others. Comparative analysis with the ancestral gastropod indicated a noteworthy rearrangement in the gene order of the *D. extinctorium* mitogenome. The Littorinimorpha exhibited four distinct rearrangement patterns, with their rearrangement similarity consistently mirroring their phylogenetic relationships. Our phylogenetic tree displayed both congruities and disparities when compared to preceding studies. Phylogenetic analyses indicated the formation of an exclusive Calyptraeoidea clade by D. extinctorium, whereas (Calyptraeoidea + (Stromboidea + (Tonnoidea + Ficoidea + Cypraeoidea) + Truncatelloidea + (Naticoidea + Littorinoidea))) constituted a distinct clade. Despite a limited number of species available for a robust phylogenetic analysis, our phylogeny garnered statistical support and aspires to provide a rational framework for future phylogenetic inquiries within the realm of Calyptraeoidea. These findings not only offer insights into the

gene arrangement characteristics within Littorinimorpha mitogenomes but also establish the groundwork for further explorations into the phylogenetic aspects of Littorinimorpha.

Author Contributions

Conceptualization: Yanwen Ma, Yingying Ye.

Data curation: Yanwen Ma, Jiji Li.

Formal analysis: Yingying Ye.

Funding acquisition: Yingying Ye.

Investigation: Biqi Zheng, Wei Meng.

Methodology: Yanwen Ma.

Project administration: Yingying Ye, Biqi Zheng.

Resources: Biqi Zheng.

Software: Yanwen Ma.

Supervision: Kaida Xu.

Validation: Kaida Xu.

Visualization: Yanwen Ma.

Writing – original draft: Yanwen Ma.

Writing – review & editing: Yingying Ye, Jiji Li.

References

1. Ballard JWO, Whitlock MC. The incomplete natural history of mitochondria. Mol Ecol. 2004 Apr; 13(4): 729-744. https://doi.org/10.1046/j.1365-294x.2003.02063.x.

2. Gissi C, Iannelli F, Pesole G. Evolution of the mitochondrial genome of Metazoa as exemplified by comparison of congeneric species. Heredity. 2008 Oct; 101(4): 301-320. https://doi.org/10.1038/hdy.2008.62.
3. Kurabayashi A, Sumida M, Yonekawa H, Glaw F, Vences M, Hasegawa M. Phylogeny, recombination, and mechanisms of stepwise mitochondrial genome reorganization in mantellid frogs from Madagascar. Mol Biol Evol. 2008 May; 25(5): 874-891. https://doi.org/10.1093/molbev/msn031.
4. Saccone C, Giorgi D, Gissi C, Pesole G, Reyes A. Evolutionary genomics in Metazoa: the mitochondrial DNA as a model system. *Gene*. 1999 Sep; 238(1): 195-209. https://doi.org/10.1016/s0378-1119(99)00270-x.
5. Ye YY, Wu CW, Li JJ. Genetic population structure of *Macridiscus multifarius* (Mollusca: Bivalvia) on the basis of mitochondrial markers: strong population structure in a species with a short planktonic larval stage. PLoS One. 2015 Dec; 10(12): e0146260. https://doi.org/10.1371/journal.pone.0146260
6. Zhang Y, Gong L, Lu XT, Jiang LH, Liu BJ, Liu LQ, et al. Gene rearrangements in the mitochondrial genome of *Chiromantes eulimene* (Brachyura: Sesarmidae) and phylogenetic implications for Brachyura. Int J Biol Macromol. 2020 Nov; 162: 704-714. https://doi.org/10.1016/j.ijbiomac.2020.06.196.
7. Kumar V, Tyagi K, Chakraborty R, Prasad P, Kundu S, Tyagi I, et al. The complete mitochondrial genome of endemic giant tarantula, *Lyrognathus crotalus* (Araneae: Theraphosidae) and comparative analysis. Sci Rep. 2020 Jan; 10(1): 74. https://doi.org/10.1038/s41598-019-57065-8.
8. Ruan HT, Li M, Li ZH, Huang JJ, Chen WY, et al. Comparative analysis of complete mitochondrial genomes of three gerres fishes (Perciformes: Gerreidae) and primary exploration of their evolution history. Int J Mol Sci. 2020 Mar; 21(5): 1874. https://doi.org/10.3390/ijms21051874.
9. Yang Y, Li Q, Kong LF, Yu H. Mitogenomic phylogeny of *Nassarius* (Gastropoda: Neogastropoda). Zool Scr. 2019 Feb 25; 48(3): 302-312. https://doi.org/10.1111/zsc.12343.
10. Yang Y, Liu HY, Qi L, Kong LF, Li Q. Complete mitochondrial genomes of two toxin-accumulated Nassariids (Neogastropoda: Nassariidae: *Nassarius*) and their implication for phylogeny. Int J Mol Sci. 2020 May 17; 21(10): 3545. https://doi.org/10.3390/ijms21103545.
11. Lee SY, Lee HJ, Kim YK. Comparative analysis of complete mitochondrial genomes with Cerithioidea and molecular phylogeny of the freshwater snail, *Semisulcospira gottschei* (Caenogastropoda, Cerithioidea). Int J Biol Macromol. 2019 Aug 15; 135: 1193-1201. https://doi.org/10.1016/j.ijbiomac.2019.06.036.
12. Knudsen J. Observation on *Calyptraea extinctorium* Lamrck, 1822 (Prosobranchia: Calyptraeidae) from Hong Kong. In The Marine Flora and Fauna of Hong Kong and Southern China IV, Morton B ed.; Hong Kong University Press, 1997; pp. 371–380. http://www.jstor.org/stable/j.ctt2jc68s.30
13. Teso V, Penchaszadeh PE. Development of the gastropod *Trochita pileus* (Calyptraeidae) in the sub-Antarctic Southwestern Atlantic. Polar Biol. 2018 Oct 19; 42: 171-178. https://doi.org/10.1007/s00300-018-2412-4.
14. Holtheuer J, Aldea C, Schories D, Gallardo CS. The natural history of Calyptraeaaurita (Reeve, 1859) from Southern Chile (Gastropoda, Calyptraeidae). Zookeys. 2018 Nov 21; 798: 1-22. https://doi.org/10.3897/zookeys.798.25736.
15. Cledón M, Nuñez JD, Ocampo EH, Sigwart JD. Sexual traits plasticity of the potentially invasive limpet *Bostrycapulus odites* (Gastropoda: Calyptraeidae) within its natural distribution in South America. Mar Ecol. 2015 Dec 9; 37(2): 433-441. https://doi.org/10.1111/maec.12329.
16. Collin R. Development, phylogeny, and taxonomy of *Bostrycapulus* (Caenogastropoda: Calyptraeidae), an ancient cryptic radiation. Zool J Linn Soc. 2005 May 10; 144(1): 75-101. https://doi.org/10.1111/j.1096-3642.2005.00162.x.
17. Cunha RL, Grande C, Zardoya R. Neogastropod phylogenetic relationships based on entire mitochondrial genomes. BMC Evol Biol. 2009 Aug 23; 9: 210. https://doi.org/10.1186/1471-2148-9-210.

18. Collin R. The effects of mode of development on phylogeography and population structure of North Atlantic *Crepidula* (Gastropoda: Calyptraeidae). Mol Ecol. 2001 Sep; 10(9): 2249-2262. https://doi.org/10.1046/j.1365-294x.2001.01372.x.
19. Bouchet P, Rocroi JP. Classification and nomenclator of gastropod families. Malacologia. 2005 Jan**;** 47(1-2): 1-397.
20. Colgan DJ, Ponder WF, Beacham E, Macaranas J. Molecular phylogenetics of Caenogastropoda (Gastropoda: Mollusca). Mol Phylogenet Evol. 2007 Mar**;** 42(3): 717-737. https://doi.org/10.1016/j.ympev.2006.10.009.
21. Osca D, Templado J, Zardoya R. Caenogastropod mitogenomics. Mol Phylogenet Evol. 2015 Dec; 93: 118-128. https://doi.org/10.1016/j.ympev.2015.07.011.
22. Zhao QP, Zhang SH, Deng ZR, Jiang MS, Nie P. Conservation and variation in mitochondrial genomes of gastropods *Oncomelania hupensis* and *Tricula hortensis*, intermediate host snails of Schistosoma in China. Mol Phylogenet Evol. 2010 Oct; 57(1): 215-226. https://doi.org/10.1016/j.ympev.2010.05.026.
23. Wang QZ, Liu HY, Yue C, Xie X, Li DC, Liang M, et al. Characterization of the complete mitochondrial genome of *Ficus variegata* (Littorinimorpha: Ficidae) and molecular phylogeny of Caenogastropoda. Mitochondrial DNA B Resour. 2021 Mar 19; 6(3): 1126-1128. https://doi.org/10.1080/23802359.2021.1901628.
24. Frank R. Recognition of the superfamily Ficoidea Meek, 1864 and definition of the Thalassocyonidae fam. nov. (Gastropoda). Zool Jahrb Abt Syst. 1994; 121: 457-474.
25. Jiang DH, Zheng XD, Zeng XQ, Kong LF, Li Q. The complete mitochondrial genome of *Harpago chiragra* and *Lambis lambis* (Gastropoda: Stromboidea): implications on the Littorinimorpha phylogeny. Sci Rep. 2019 Nov 27; 9(1): 17683. https://doi.org/10.1038/s41598-019-54141-x.
26. Aljanabi S, Martinez I. Universal and rapid salt-extraction of high quality genomic DNA for PCR-based techniques. Nucleic Acids Res. 1997 Nov 15; 25(22): 4692‑4693. https://doi.org/10.1093/nar/25.22.4692.
27. Dierckxsens N, Mardulyn P, Smits G. NOVOPlasty: de novo assembly of organelle genomes from whole genome data. Nucleic Acids Res. 2017 Feb 28; 45(4): e18. https://doi.org/10.1093/nar/gkw955.
28. Bernt M, Donath A, Jühling F, Externbrink F, Florentz C, Fritzsch G, et al. MITOS: improved de novo metazoan mitochondrial genome annotation. Mol Phylogenet Evol. 2012 Nov; 69(2): 313‑319. https://doi.org/10.1016/j.ympev.2012.08.023.
29. Altschul SF, Madden TL, Schäffer AA, Zhang J, Zhang Z, Miller W, et al. Gapped BLAST and PSI-BLAST: a new generation of protein database search programs. Nucleic Acids Res. 1997 Sep 1; 25(17): 3389‑3402. https://doi.org/10.1093/nar/25.17.3389.
30. Grant JR, Stothard P. The CGView Server: a comparative genomics tool for circular genomes. Nucleic Acids Res. 2008 Jul 1; 36(Web Server issue): W181‑184. https://doi.org/10.1093/nar/gkn179.
31. Kumar S, Stecher G, Tamura K. MEGA7: Molecular evolutionary genetics analysis version 7.0 for bigger datasets. Mol Biol Evol. 2016 Jul; 33(7): 1870‑1874. https://doi.org/10.1093/molbev/msw054.
32. Xia X, Xie Z. DAMBE: software package for data analysis in molecular biology and evolution. J Hered. 2001 Jul-Aug; 92(4): 371‑373. https://doi.org/10.1093/jhered/92.4.371.
33. Perna NT, Kocher TD. Patterns of nucleotide composition at fourfold degenerate sites of animal mitochondrial genomes. J Mol Evol. 1995 Sep; 41(3): 353‑358. https://doi.org/10.1007/BF00186547.
34. Huelsenbeck JP, Ronquist F. MRBAYES: Bayesian inference of phylogenetic trees. Bioinformatics. 2001 Aug; 17(8): 754‑755. https://doi.org/10.1093/bioinformatics/17.8.754.
35. Nguyen L, Schmidt HA, Haeseler VA, Minh BQ. IQ-TREE: a fast and effective stochastic algorithm for estimating maximum-likelihood phylogenies. Mol Biol Evol. 2015 Jan; 32(1): 268‑274. https://doi.org/10.1093/molbev/msu300.

36. Ronquist F, Teslenko M, Ayres DL, Darling A, Höhna S, Larget B, et al. MrBayes 3.2: efcient Bayesian phylogenetic inference and model choice across a large model space. Syst. Biol. 2012 May; 61(3): 539‑542. https://doi.org/10.1093/sysbio/sys029.
37. Nylander JA, Ronquist F, Huelsenbeck JP, Nieves-Aldrey JL. Bayesian phylogenetic analysis of combined data. Syst Biol. 2004 Feb; 53(1): 47‑67. https://doi.org/10.1080/10635150490264699.
38. Posada D, Crandall KA. MODELTEST: testing the model of DNA substitution. Bioinformatics. 1998; 14(9): 817‑818. https://doi.org/10.1093/bioinformatics/14.9.817.
39. FigTree, version 1.4.3. Available online: http://tree.bio.ed.ac.uk/software/figtree/ (accessed on 1 July 2016)
40. Miao J, Feng JT, Liu XJ, Yan CR, Ye YY, Li JJ, et al. Sequence comparison of the mitochondrial genomes of five brackish water species of the family Neritidae: Phylogenetic implications and divergence time estimation. Ecol Evol. 2022 Jun 6; 12(6): e8984. https://doi.org/10.1002/ece3.8984
41. Xu MH, Gu ZQ, Huang J, Guo BY, Jiang LH, Xu KD, et al. The Complete Mitochondrial Genome of *Mytilisepta virgata* (Mollusca: Bivalvia), Novel Gene Rearrangements, and the Phylogenetic Relationships of Mytilidae. Genes. 2023 Apr 13; 14(4): 910. https://doi.org/10.3390/genes14040910.
42. Xu MH, Li JJ, Guo BY, Xu KD, Ye YY, Yan XJ. Insights into the Deep Phylogeny and Novel Gene Rearrangement of Mytiloidea from Complete Mitochondrial Genome. Biochem Genet. 2023 Oct; 61(5): 1704-1726. https://doi.org/10.1007/s10528-023-10338-4.
43. Wang Y, Yang Y, Liu Y, Kong LF, Yu H, Liu SK, et al. Phylogeny of Veneridae (Bivalvia) based on mitochondrial genomes. Zool Scr. 2020 Oct 25; 50(1): 58-70. https://doi.org/10.1111/zsc.12454
44. Yuan Y, Li Q, Yu H, Kong LF. The complete mitochondrial genomes of six heterodont bivalves (Tellinoidea and Solenoidea): variable gene arrangements and phylogenetic implications. PLoS One. 2012; 7(2): e32353. https://doi.org/10.1371/journal.pone.0032353.
45. Lü JY, Dong XL, Li JJ, Ye YY. Novel gene re-arrangement in the mitochondrial genome of *Pisidia serratifrons* (Anomura, Galatheoidea, Porcellanidae) and phylogenetic associations in Anomura. Biodivers Data J. 2023 Feb; 11(4): e96231. https://doi.org/10.3897/BDJ.11.e96231.
46. Sun S, Sha Z, Wang Y. The complete mitochondrial genomes of two vent squat lobsters, *Munidopsis lauensis* and *M. verrilli*: Novel gene arrangements and phylogenetic implications. Ecol Evol. 2019 Sep 30; 9(22): 12390‑12407. https://doi.org/10.1002/ece3.5542.
47. Yu Y, Kong L, Li Q. Mitogenomic phylogeny of Muricidae (Gastropoda: Neogastropoda). Zool Scr. 2023 Apr 24; 52(4): 413-425. https://doi.org/10.1111/zsc.12598
48. Yang HR, Zhang JE, Luo H, Luo MZ, Guo J, Deng ZX, et al. The complete mitochondrial genome of the mudsnail *Cipangopaludina cathayensis* (Gastropoda: Viviparidae). *Mitochondrial. DNA. A. DNA.* Mapp Seq Anal. 2016 May; 27(3): 1892-1894. https://doi.org/10.3109/19401736.2014.971274.
49. Tan MH, Gan HM, Lee YP, Bracken-Grissom H, Chan TY, Miller AD, et al. Comparative mitogenomics of the Decapoda reveals evolutionary heterogeneity in architecture and composition. Sci Rep. 2019 Jul 24; 9(1): 10756. https://doi.org/10.1038/s41598-019-47145-0.
50. Kilpert F, Held C, Podsiadlowski L. Multiple rearrangements in mitochondrial genomes of Isopoda and phylogenetic implications. Mol Phylogenet Evol. 2012 Jul; 64: 106-117. https://doi.org/10.1016/j.ympev.2012.03.013.
51. Bernt M, Bleidorn C, Braband A, Dambach J, Donath A, Fritzsch G, et al. A comprehensive analysis of bilaterian mitochondrial genomes and phylogeny. Mol Phylogenet Evol. 2013 Nov; 69(2): 352-364. https://doi.org/10.1016/j.ympev.2013.05.002.

52. Xu W, Jameson D, Tang B, Higgs PG. The relationship between the rate of molecular evolution and the rate of genome rearrangement in animal mitochondrial genomes. J Mol Evol. 2006 Sep; 63(3): 375-392. https://doi.org/10.1007/s00239-005-0246-5.
53. Xu T, Qi L, Kong LF, Li Q. Mitogenomics reveals phylogenetic relationships of Patellogastropoda (Mollusca, Gastropoda) and dynamic gene rearrangements. Zool Scr. 2022 Dec 20; 51(2): 147-160. https://doi.org/10.1111/zsc.12524
54. Zhang Y. Mitochondrial genome rearrangement of Sesarmidae species and its phylogenetic implication. Zhejiang Ocean University, Zhejiang, 2022. https://doi.org/10.27747/d.cnki.gzjhy.2022.000441.
55. Uribe JE, Kano Y, Templado J, Zardoya R. Mitogenomics of Vetigastropoda: insights into the evolution of pallial symmetry. Zool Scr. 2016, 45, 145-159. https://doi.org/10.1111/zsc.12146.
56. Osca D, Templado J, Zardoya R. The mitochondrial genome of *Ifremeria nautilei* and the phylogenetic position of the enigmatic deep-sea Abyssochrysoidea (Mollusca: Gastropoda). Gene. 2014 Sep 1; 547(2): 257-266. https://doi.org/10.1016/j.gene.2014.06.040.
57. Choi EH, Hwang UW. The complete mitochondrial genome of an endangered triton snail *Charonia lampas* (Littorinimorpha: Charoniidae) from South Korea. Mitochondrial DNA B Resour. 2021 Mar 18; 6(3): 956-958. https://doi.org/10.1080/23802359.2021.1889416.
58. Zhong SP, Huang LH, Huang GQ, Liu YH, Wang WX. The first complete mitochondrial genome of mammilla from *Mammilla mammata* (Littorinimorpha: Naticidae). Mitochondrial DNA B Resour. 2019 Dec 12; 5(1): 96-97. https://doi.org/10.1080/23802359.2019.1698350.
59. Irwin AR, Strong EE, Kano Y, Harper EM, Williams ST. Eight new mitogenomes clarify the phylogenetic relationships of Stromboidea within the caenogastropod phylogenetic framework. Mol Phylogenet Evol. 2021 May; 158: 107081. https://doi.org/10.1016/j.ympev.2021.107081.

Manuscript Click here to access/download;Figure;Fig.1.tif

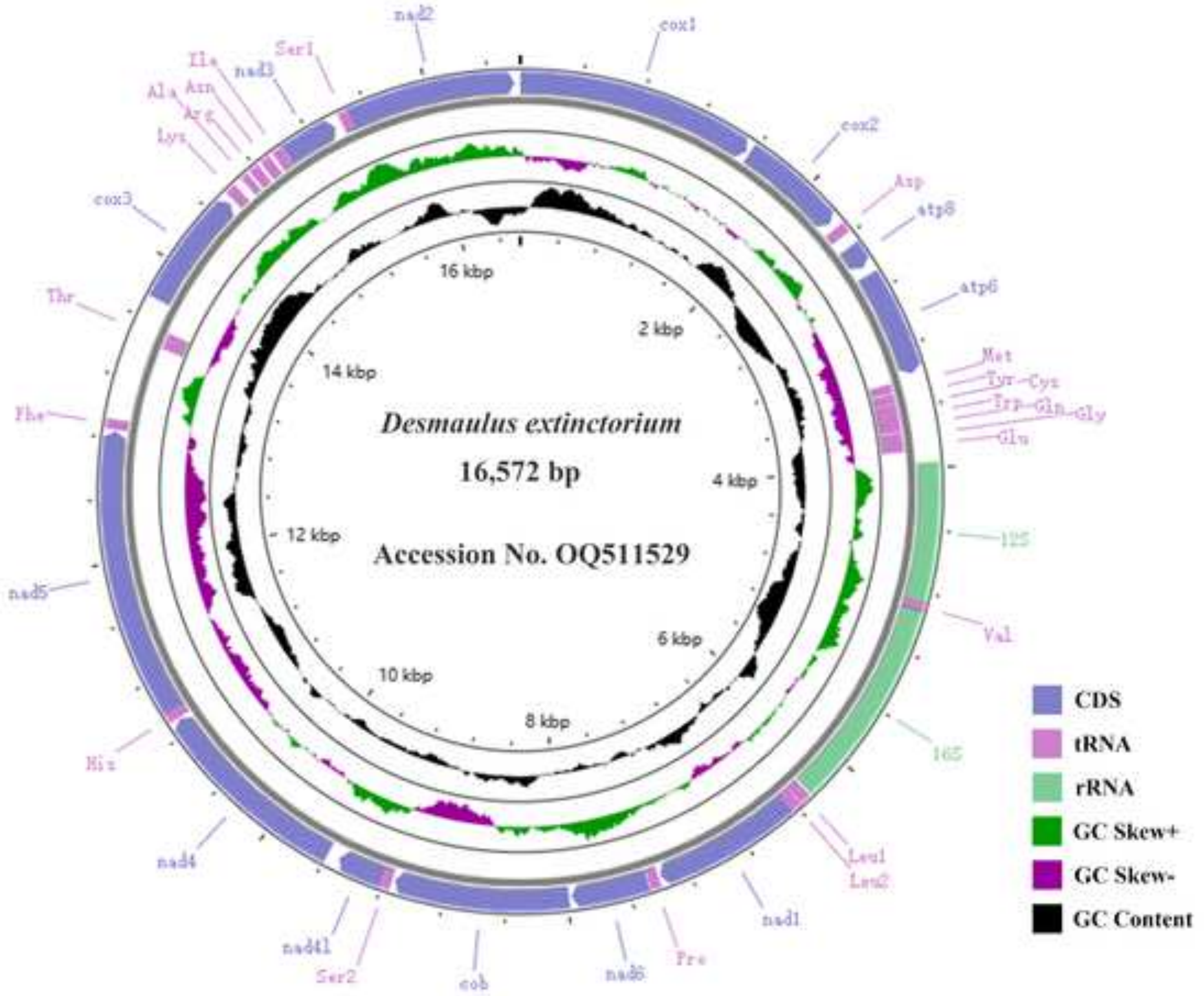

Manuscript

Click here to access/download;Figure;Fig. 2.tif

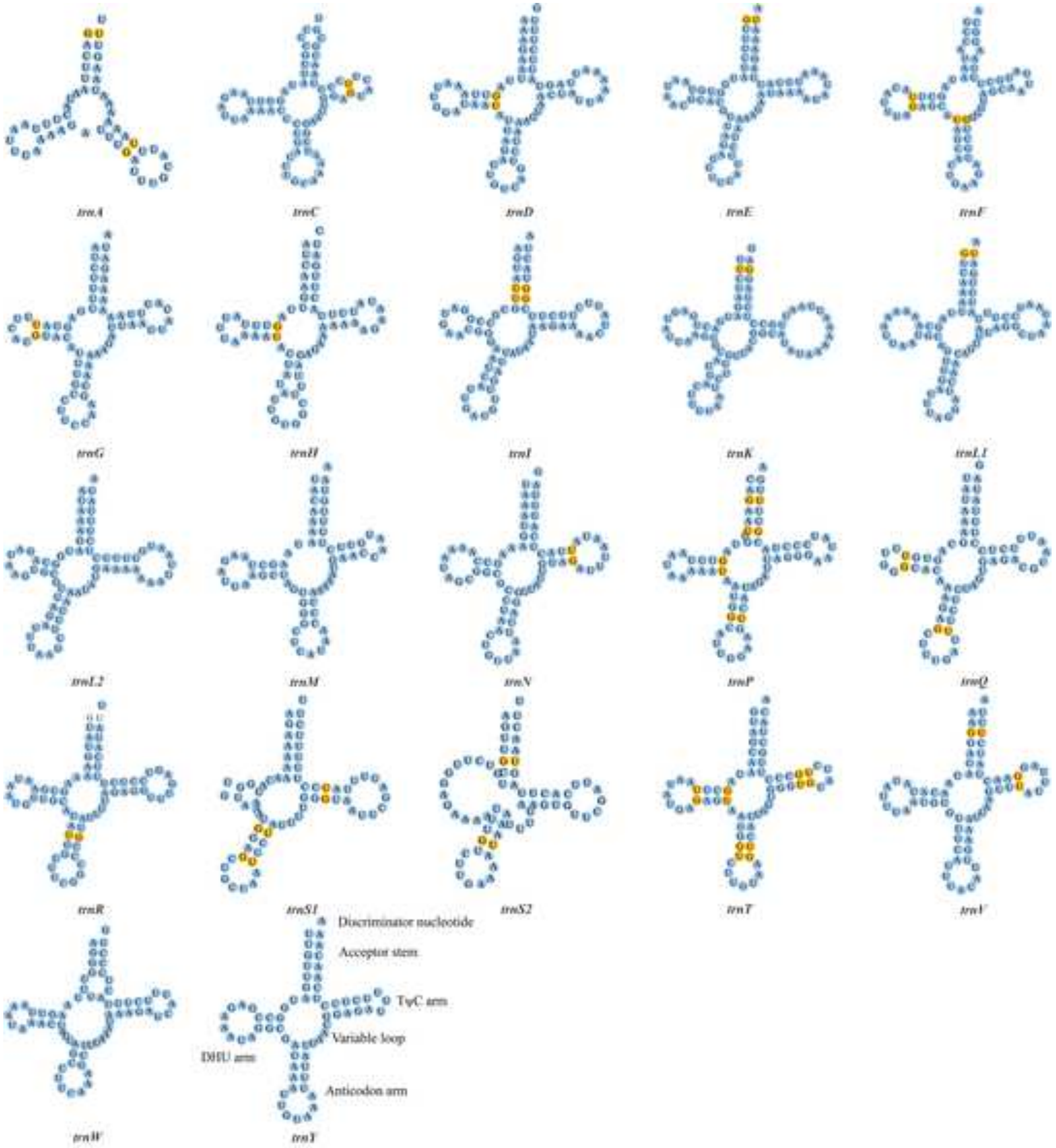

Manuscript Click here to access/download;Figure;Fig. 3.tif

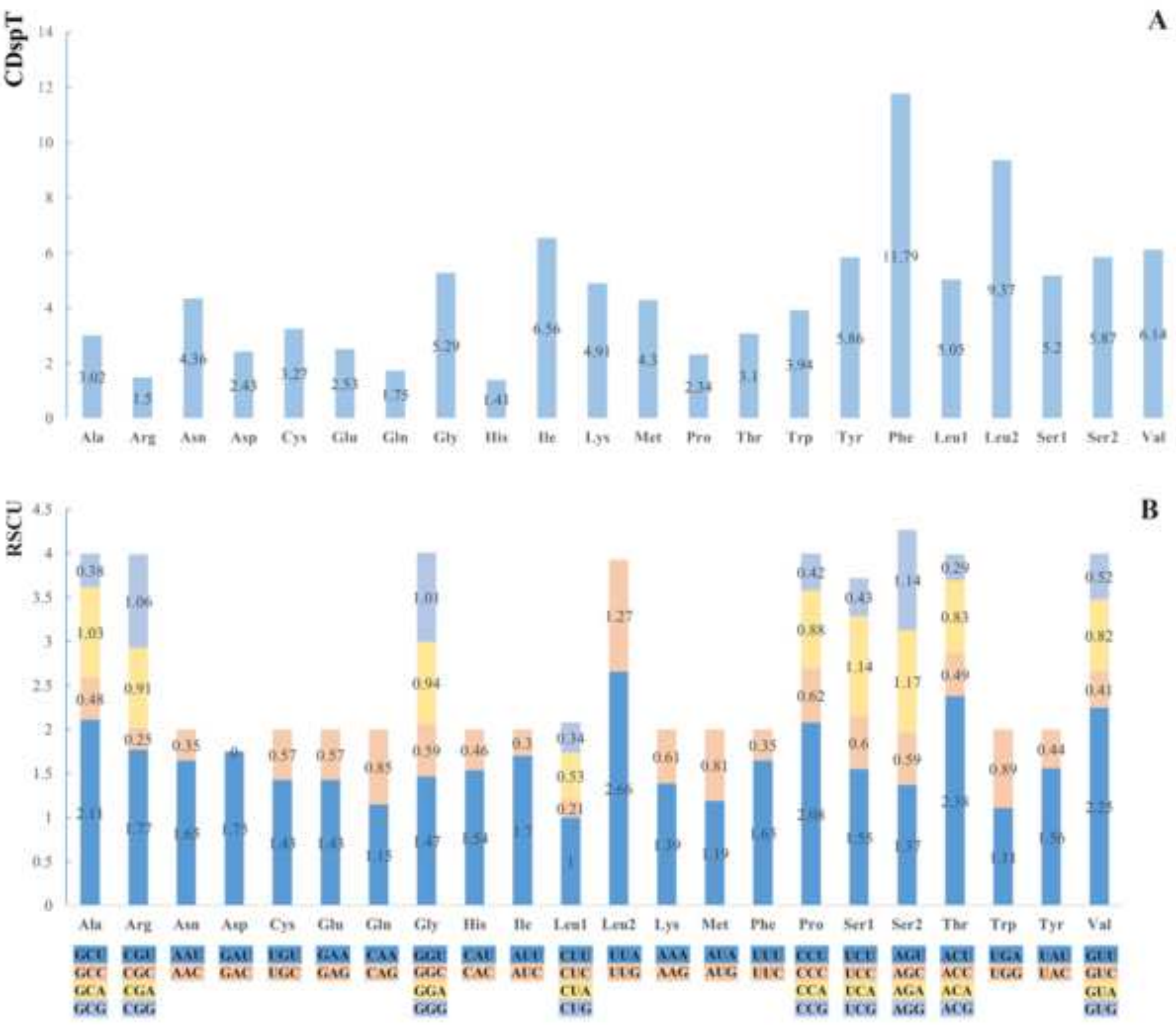

Manuscript

Click here to access/download;Figure;Fig. 4.tif

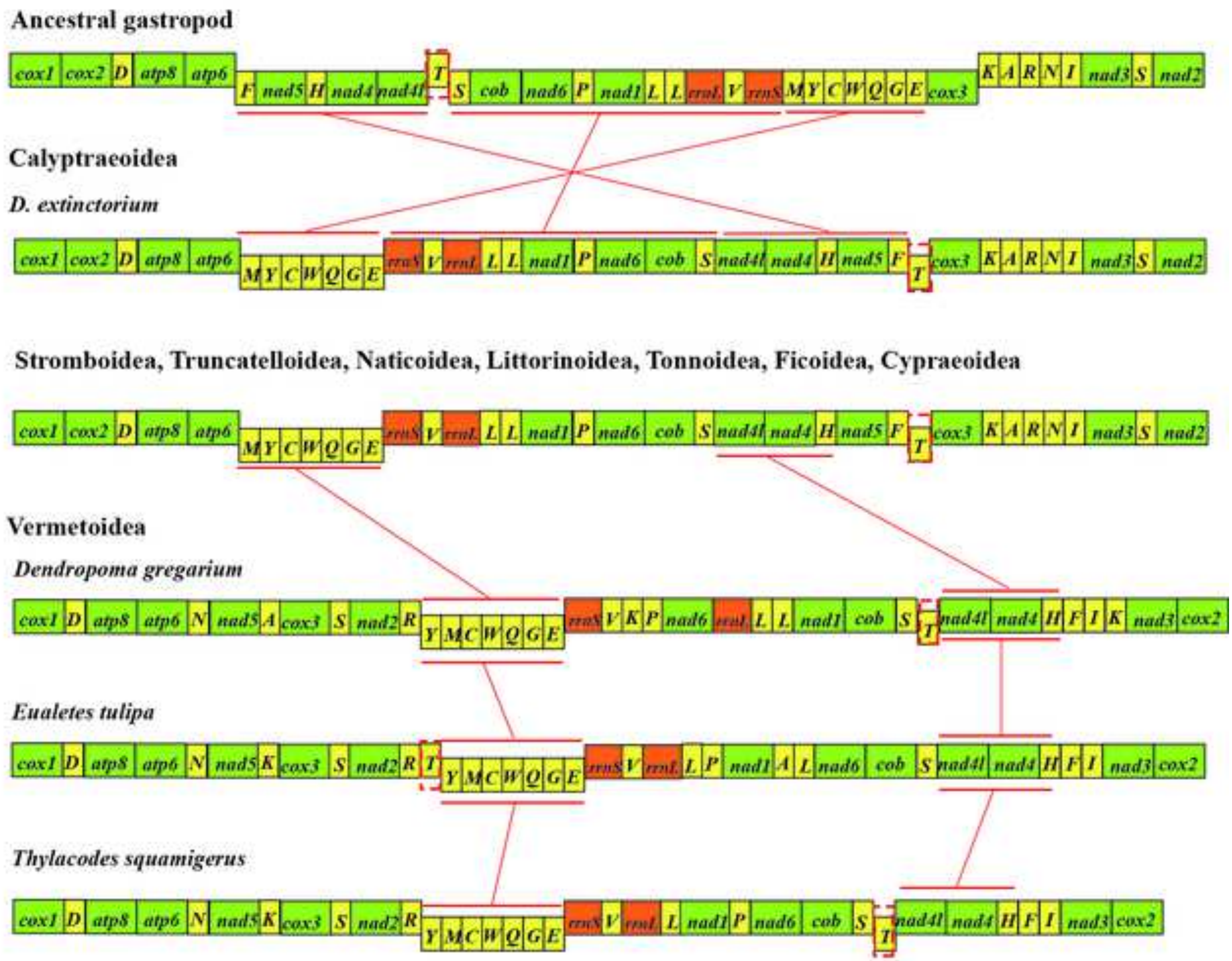

Manuscript Click here to access/download;Figure;Fig. 5.tif

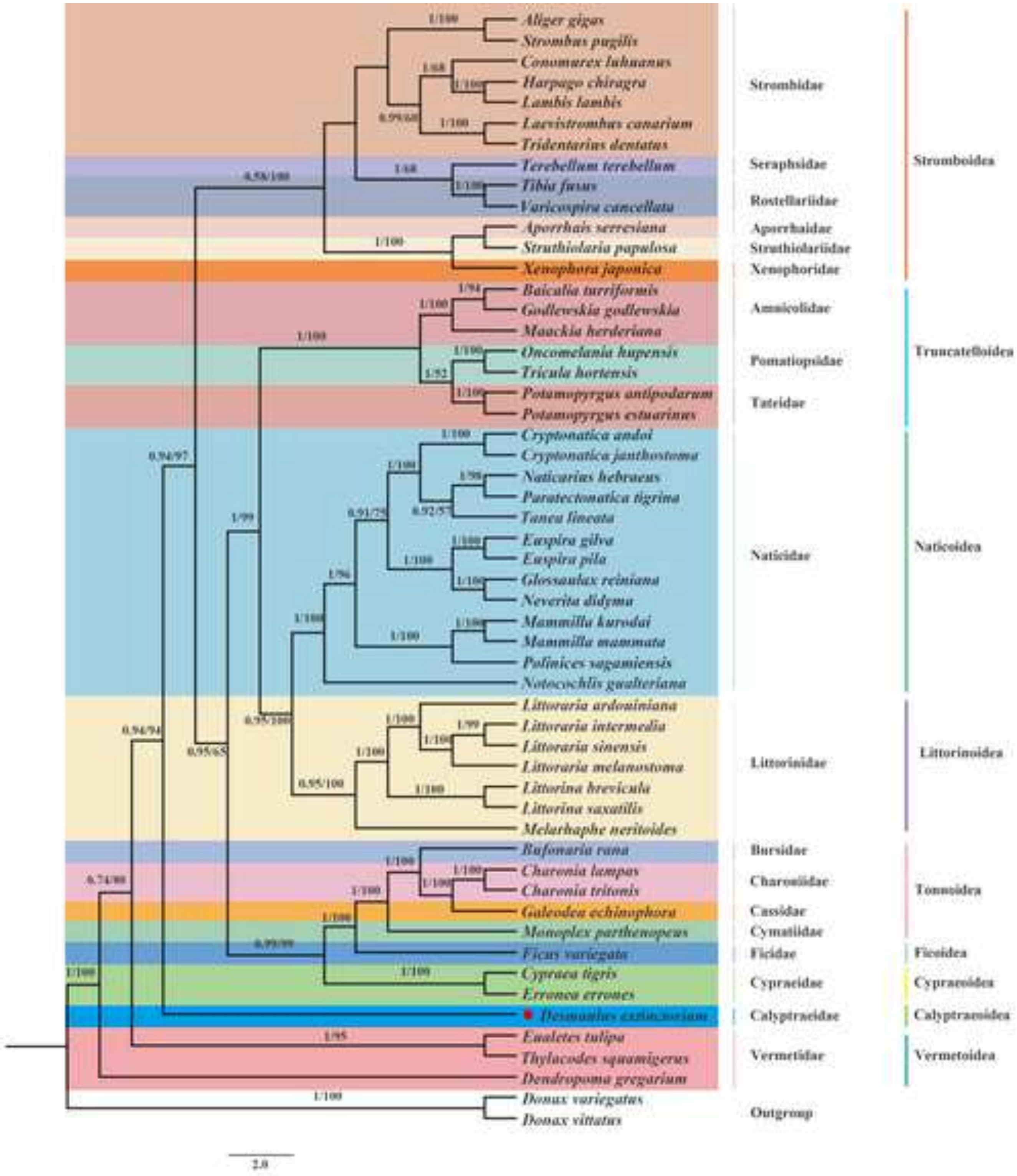

Supplement: Attachment — Submitted filename: PONE-D-23-34396_reviewed.pdf [file pone.0301389.s001.pdf]
