# Supplementary material for: Characterization of the complete mitochondrial genome of Desmaulus extinctorium (Littorinimorpha, Calyptraeoidea, Calyptraeidae) and molecular phylogeny of Littorinimorpha
Source: PLoS One. 2024 Mar 28;19(3):e0301389. doi: 10.1371/journal.pone.0301389 (PMC10977763; doi:10.1371/journal.pone.0301389)
Supplement: Attachment — Submitted filename: Response to Reviewers.docx [file pone.0301389.s002.docx]

**Dear editor and reviewers,**

We thank you for the constructive comments. We have provided detailed responses to all comments below. All revisions to the manuscript have been marked up using the “Track Changes” function in the current version. We are looking forward to your approval or further advice on improving the manuscript, if any.

**Major issues**

• A major risk in molecular studies is misidentification of the specimens that were sequenced (there are numerous examples). Therefore it is useful to add photographs of those specimens and indicate where they were collected. As many species included are based on previous or ongoing studies, can you provide the relevant literature references for each? [By the way, I see other papers, e.g. in respectable magazines like Nature, that provide less data than the current study].

Answer: All published references have been cited in Table 1. The genomes of some species have only been uploaded to GenBank for the time being, and the references have not yet been published. As Phylogenetic relationships of the Balkan Moitessieriidae (Caenogastropoda: Truncatelloidea) and Phylogeny of Strombidae (Gastropoda) Based on Mitochondrial Genomes show, some of the genomes involved also have unpublished ones.

• Add at least photographs of the specimen analysed.

Answer: The photographs of the specimen have been added in Fig1.

• Line 49 I have no idea what geological mud is, but suppose you mean siliciclastic mud (instead of carbonate mud) . Anyway, Desmaulus extinctorium is typically found on sandy substrates from low intertidal to several metres subtidally. It is a commensal of hermit crabs, living on the outside of gastropod shells inhabited by these crabs. [own observations and e.g. ref. 12 and Raven JGM (2019) Crepidula fornicata (Linnaeus, 1758) (Gastropoda: Calyptraeidae) as a hermit crab commensal in the North Sea. Nautilus 133:40–47]

Answer: The sentence has been revised.

• Line 51 – Desmaulus extinctorium is certainly not restricted to southern China and Hong Kong but has a wide distribution in the Indo-West Pacific.

Answer: The sentence has been revised.

• Please ensure all references in the text make sense. Either refer to last name, last name et al. or [number]. For example line 55 Regina without any reference number, line 72 Regina et al [17] – referring to the first name of the first author of Cunha et al. [17]. Line 58 Rachel - referring to the first name of Collin [16]. One can only find this by going to the papers referred to. Ditto Line 301 - Alison R. Irwin --> Irwin et al. Please check throughout.

Answer: These details have been reviewed and modified.

• Line 127-135 There is no comment whether any effort weas made to ensure the 51 mitogenomes obtained from GenBank are based on correct identifications. Whilst checking GenBank and underlying studies it is evident the Stromboidea and Naticidae data originate from respectable studies, it would be useful to explicitly state these Littorinimorpha species were analysed by specialists in each group, adding references for each species in Table 1. In many molecular (and other) studies some or a substantial number of the specimens studied have been misidentified. Where authors figure the specimens and list their provenance at least their identity can be verified.

Answer: All published references have been cited in Table 1. The genomes of some species have only been uploaded to GenBank for the time being, and the references have not yet been published. As Phylogenetic relationships of the Balkan Moitessieriidae (Caenogastropoda: Truncatelloidea) and Phylogeny of Strombidae (Gastropoda) Based on Mitochondrial Genomes show, some of the genomes involved also have unpublished ones.

• Line 295-297 – You present this as your conclusion, but for example the species/molecular data and resulting clades for the Stromboidea are virtually the same as used in the previous study [59], only the tree is presented slightly differently. You could be much briefer and indicate that, as expected, your study confirms the conclusions of [59].

Answer: This part has been revised.

• The Naticidae genomes were all taken from Liu et al. (2020) Mitogenomic phylogeny of the Naticidae (Gastropoda: Littorinimorpha) reveals monophyly of the Polinicinae. – but it is not in the references. As is to be extected, the outcome is similar, with some species reversed in the figure. In Liu et al. the key finding (illustrated in their fig. 2) is that the two subfamilies (Naticinae with calcareous operculum and Polinicinae with corneous operculum) are confirmed, but Notocochlis gualtieriana and Notocochlis sp. with calcareous operculum) form a separate clade, making the Naticinae paraphyletic. In your figure 5 Notocochlis is placed within the group Liu et al. consider Polinicinae. If you present it this way you should at least explain why and what the implications are – or not repeat all these data and just select one or two Naticidae.

Answer: In our figure 5, Notocochlis is not placed within Polininae; instead, it remains a species within Naticinae. The divergence arises because our study selected a broader range of Naticidae species genomes from NCBI to construct the phylogenetic tree. Consequently, the branch of Naticinae is divided into two parts, indicating that Naticinae lacks monophyly. In Liu et al.'s study, there is no explicit confirmation of the monophyly of Naticinae either. Subsequent investigations may require additional genomic data from more Naticidae species to address this issue. Therefore, these details are not elaborated upon in the manuscript.

• Iine 297-298 “At the family level, species of each family formed a distinct clade.” – I assume you mean: Within each superfamily, each family forms a distinct clade.

Answer: The sentence has been revised.

• The larger number of species from e.g. Naticidae and Littorinidae does not add to understanding placement of Desmaulus within Littorinimorpha. More useful to see data for other Calyptraeidae or potentially closely related families such as Hipponicidae or Capulidae. There is ample genetic material of these in GenBank, but I do not known whether there are any with sufficient mitochondrial DNA to make a good comparison.

Answer: Due to the exclusive utilization of complete mitochondrial genomes in this study for constructing the phylogenetic tree, there is currently an absence of complete mitochondrial genome information for species within Hipponicidae in the GenBank database. For Capulidae species, there is only one complete mitochondrial genome available, uploaded in February 2024, which is insufficient for a comprehensive comparative analysis. As a result, the study does not include a phylogenetic analysis of Hipponicidae and Capulidae.

• Line 301-302 – These lines confuse what is covered in the study with what genera and species are in each family [this occurs throughout the text]. Assume you want to say: In our study Naticidae is the family of which most species have been included, representing a large number of genera. The Littorinidae are its sister group, of which a substantial number of species has been included in our study, however only representing a selection of the genera.

Answer: This part has been revised.

**Minor issues**

• Quite a bit of the text suffers from unclear formulations or details that do not really add to the storyline. Some simple editing can improve this. I have selected a few examples.

Answer: The sentence has been revised.

• Line 12 – For the purpose of augmenting the taxonomy and systematics of Calyptraeidae in the evolutionary framework of Littorinimorpha --> why not simply say: For the purpose of determining the placement (or position) of Calyptraeidae within the Littorinimorpha.

Answer: The sentence has been revised.

• Line 22-25: better say something like: D. extinctorium representing a distinct Calyptraeoidea clade. In summary, this investigation provides the first complete mitochondrial dataset for a species of the Calyptraeidae, thus providing novel insights into the phylogenetic relationships within the Littorinimorpha. (You only analysed one species within this clade, which is formed by many more in 11 genera).

Answer: The sentence has been revised.

• Line 42: Nassarius members --> Nassarius species

Answer: The word has been revised.

• Line 43: Nassariids --> nassarids

Answer: The word has been revised.

• Line 46: relationship within gastropod species ??? Do you mean relationship with other gastropod species?

Answer: The sentence has been revised.

• Line 65: family --> order; then also add subclass before Caenogastropoda – or just state that Littorinimorpha comprises 16 superfamilies as the other bit is already stated in line 50.

Answer: The sentence has been revised.

• Iine 68 - check: do you mean classification OF Littorinimorpha or classification WITHIN Littorinimorpha?

Answer: The sentence has been revised.

• Line 68: was mainly established by Bouchet [19]. ◊ was established by Bouchet & Rocroi [19].

Answer: The sentence has been revised.

• Line 79-83: quite unclear. Why not simply state: Riedel [24] established the superfamily Ficoidea, separate from the Tonnoidea, but based on the sequencing of the complete mitochondrial genome of Ficus variegata Wang et al. [23] demonstrated that it fits within the Tonnoidea.

Answer: The sentence has been revised.

• Line 80 & 81: variegate --> variegata [common error caused by the automatic spell checker]

Answer: The sentence has been revised.

• Line 82 – Riedel reclassified figs – Riedel reclassified fig shells (or even better Ficidae).

Answer: The sentence has been revised.

• Line 296 – recent families --> extant families

Answer: The word has been revised.
